# Stromal Hedgehog pathway activation by IHH suppresses lung adenocarcinoma growth and metastasis by limiting reactive oxygen species

**DOI:** 10.1038/s41388-020-1224-5

**Published:** 2020-02-27

**Authors:** Sahba Kasiri, Baozhi Chen, Alexandra N. Wilson, Annika Reczek, Simbarashe Mazambani, Jashkaran Gadhvi, Evan Noel, Ummay Marriam, Barbara Mino, Wei Lu, Luc Girard, Luisa M. Solis, Katherine Luby-Phelps, Justin Bishop, Jung-Whan Kim, James Kim

**Affiliations:** 1 Nancy B. and Jake L. Hamon Center for Therapeutic Oncology Research and Harold C. Simmons Comprehensive Cancer Center, Dallas, TX USA; 2 Department of Biological Sciences, The University of Texas at Dallas, Richardson, TX 75080 USA; 3 Department of Translational Molecular Pathology, The University of Texas MD Anderson Cancer Center, Houston, TX 77030 USA; 4 Department of Cell Biology, University of Texas Southwestern Medical Center, Dallas, TX 75390 USA; 5 Department of Pathology, University of Texas Southwestern Medical Center, Dallas, TX 75390 USA; 6 Department of Internal Medicine, University of Texas Southwestern Medical Center, Dallas, TX 75390 USA

**Keywords:** Non-small-cell lung cancer, Cancer microenvironment, Tumour angiogenesis, Cancer models, Mechanisms of disease

## Abstract

Activation of the Hedgehog (Hh) signaling pathway by mutations within its components drives the growth of several cancers. However, the role of Hh pathway activation in lung cancers has been controversial. Here, we demonstrate that the canonical Hh signaling pathway is activated in lung stroma by Hh ligands secreted from transformed lung epithelia. Genetic deletion of *Shh*, the primary Hh ligand expressed in the lung, in *Kras*^*G12D/+*^*;Trp53*^*fl/fl*^ autochthonous murine lung adenocarcinoma had no effect on survival. Early abrogation of the pathway by an anti-SHH/IHH antibody 5E1 led to significantly worse survival with increased tumor and metastatic burden. Loss of IHH, another Hh ligand, by in vivo CRISPR led to more aggressive tumor growth suggesting that IHH, rather than SHH, activates the pathway in stroma to drive its tumor suppressive effects—a novel role for IHH in the lung. Tumors from mice treated with 5E1 had decreased blood vessel density and increased DNA damage suggestive of reactive oxygen species (ROS) activity. Treatment of *Kras*^*G12D/+*^*;Trp53*^*fl/fl*^ mice with 5E1 and N-acetylcysteine, as a ROS scavenger, decreased tumor DNA damage, inhibited tumor growth and prolonged mouse survival. Thus, IHH induces stromal activation of the canonical Hh signaling pathway to suppress tumor growth and metastases, in part, by limiting ROS activity.

## Introduction

Lung cancer is the leading cause of cancer-related mortality in the U.S. and the world [1] with 5-year survival of <5% for patients with metastatic disease [2]. Non-small cell lung cancer (NSCLC) accounts for ~85% of lung cancers, of which, adenocarcinoma is the most common subtype of NSCLC (http://seer.cancer.gov/csr/1975_2007/results_merged/sect_15_lung_bronchus.pdf). *KRAS* mutations are the most common oncogenic driver mutations and occur in ~30% of lung adenocarcinoma (LAD) [3]. Currently, specific targeted therapies for mutant KRAS LAD are not available in the clinic.

The Hedgehog (Hh) signaling pathway is critical for embryonic development, tissue homeostasis, and cancer [4]. The pathway primarily operates in a paracrine manner in which a secreted Hh ligand (Sonic Hh (SHH), Indian Hh (IHH), and Desert Hh (DHH) in mammals) binds to Patched (PTCH), a 12-pass transmembrane protein, to relieve its basal inhibition of Smoothened (SMO), a seven-pass transmembrane protein. SMO activation leads to activation and nuclear localization of the glioma-associated oncogene 2 (GLI2) transcription factor to initiate the transcription of target genes, including *PTCH*, glioma-associated oncogene 1 (*GLI1)*, and Hh interacting protein *(HHIP)* [4].

Aberrant activation of the Hh signaling pathway by mutations in pathway components such as *PTCH*, *SUFU*, *SMO*, and amplifications of *GLI1* and *GLI2* drive tumor growth in basal cell carcinoma (BCC) [5], medulloblastoma [6], keratocystic odontogenic tumors [7, 8], meningioma [9–11], and ameloblastoma [12]. Vismodegib [13], sonidgeib [14], and glasdegib [15], potent SMO antagonists, have been approved by the FDA for clinical use [16–18].

Mutations of Hh pathway components are rare in sporadic epithelial tumors of endodermal origin such as lung, pancreas, gut and prostate cancers. It was proposed that these cancers recapitulated development by secreting Hh ligands from the tumor epithelia to activate the pathway in stromal cells that, in turn, secreted factors instrumental for tumor initiation and growth [19]. However, recent data suggest that paracrine activation of stroma by Hh ligands promotes fibroblast expansion and restrains tumor growth early in the tumorigenic process. Inhibition of stromal pathway activation led to accelerated tumor growth with more aggressive, higher grade tumors [20–25].

In lung cancers, a variety of roles for the Hh signaling pathway has been reported. In autochthonous mouse models of small cell lung cancer (SCLC), overexpression of SHH or SMOM2, a constitutively active mutant, in *Rb*^*−/*−^*;Trp53*^*−/*−^ cancer cells promoted tumor proliferation [26, 27], loss of SMO led to significantly decreased tumor formation, and treatment with sonidegib inhibited tumor growth of chemotherapy-resistant SCLC in vivo [26]. However, a phase III clinical trial showed no benefit of adding vismodegib to standard chemotherapy in treatment-naïve SCLC patients [28]. For NSCLC, distinct modes of action have been reported for the Hh signaling pathway. In lung squamous cell carcinoma (LSCC) tumor-spheres [29], SOX2 activation induced upregulation of Hh acyltransferase (HHAT) [30], a critical component that palmitoylates Hh ligands, and induced autocrine pathway activation to drive growth of LSCC tumor-spheres but not bulk LSCC cells nor LAD tumor-spheres [29]. Alternatively, in *PIK3CA-*amplified LSCC, PI3K-mTOR pathway activation led to non-canonical GLI1 expression independent of the Hh pathway [31]. GLI1 activation drove LSCC growth and treatment with combinatorial PI3K and GLI1 antagonists induced tumor regression in vivo [31]. In LAD tumor-spheres and cell lines, paracrine SHH from LAD epithelia activated the pathway in stroma to express VEGF that in turn, bound to NRP2 receptor to activate the MAPK pathway and express GLI1 in a non-canonical manner [32]. Given these varied results of the pathway’s role and modes of action in lung cancers and other solid tumors, we tested the role of paracrine Hh pathway activation in LAD tumorigenesis and growth in autochthonous mutant *Kras*^*G12D/+*^*;Trp53*^*fl/fl*^ mouse model of LAD.

## Results

### SHH ligand is expressed in lung adenocarcinoma and activates stromal Hh pathway by a paracrine mechanism

**Fig. 1:**
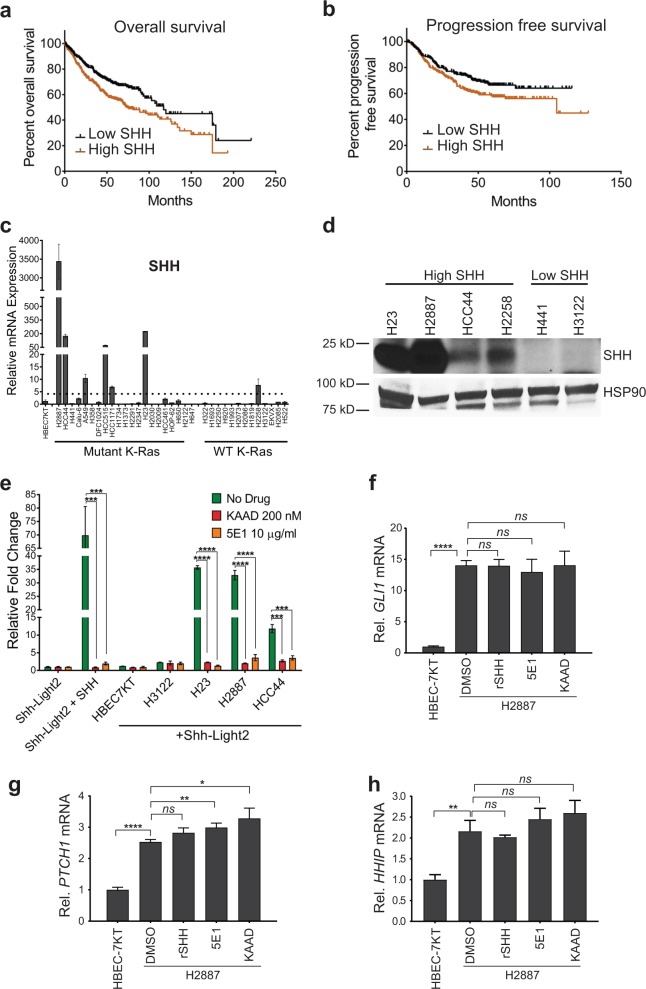
SHH in human lung adenocarcinoma. **a**, **b** Survival analyses of lung adenocarcinoma patients with high- and low*-SHH* mRNA expression from Kaplan–Meier Plotter database [36]. *n* = 720 patients. High and low mRNA expression is relative to median expression. **a** Kaplan–Meier plots by univariate analysis of overall survival (*P* = 0.0006) and (**b**) progression-free survival (*P* = 0.044) of lung adenocarcinoma patients are shown. **c** Expression of *SHH* mRNA as measured by qPCR relative to a normal bronchial epithelial cell line (HBEC7-KT). Dashed line represents 4× expression relative to HBEC7-KT. **d** Immunoblot of active N-terminal SHH of high and low SHH-expressing lung cancer cell lines from **c**. **e** Relative Hh pathway activity of Shh-Light2 fibroblasts with an 8×-GLI-luciferase reporter is shown. Shh-Light2 cells were co-cultured with low SHH-expressing HBEC7-KT normal lung epithelial cell line, low SHH-expressing H3122 LAD cell line, and high SHH-expressing H23, H2887 and HCC44 LAD cell lines. Cell lines were treated with control vehicle, KAAD-cyclopamine 200 nM, and 5E1 10 μg/ml. **f**–**h** Expression of Hh-pathway target genes in high *SHH* mRNA expressing H2887cell line. H2887 cells were treated with control vehicle (DMSO), recombinant SHH (rSHH) 1 μg/ml, KAAD-cyclopamine 300 nM, or 5E1 10 μg/ml. Expression of (**f**) *GLI1*, (**g**) *PTCH1*, and (**h**) *HHIP* were measured by qPCR relative to HBEC7-KT cell line. All qPCR data represent mean of triplicates ± SD. *P < 0.05, **P < 0.01, ***P < 0.001, ****P < 0.0001. *ns* not significant.

We evaluated the impact of SHH expression on LAD patients as SHH is the primary Hh ligand critical for lung development [33] and adult lung airway homeostasis [34]. SHH expression and activity has also been reported in lung cancers [26, 27, 30, 32, 35]. We assessed the impact of high *SHH* mRNA expression in LAD patients in the Kaplan–Meier Plotter (KM-Plotter; [36]) database that aggregates Affymetrix microarray mRNA expression data with clinical annotation. From 720 LAD patients and using median expression as the cutoff, a univariate Cox regression analysis of high *SHH* mRNA expression significantly correlated with worse overall survival (*P* = 0.0006; Fig. 1a) and progression-free survival (*P* = 0.044; Fig. 1b). These results were corroborated when stage, gender, and smoking history were accounted for in multivariate analyses for overall survival (Supplementary Fig. 1a) but not in progression-free survival (Supplementary Fig. 1b). We then surveyed 34 human LAD cell lines for Hh ligand expression by qPCR (Fig. 1c). Mutant *KRAS* cell lines were sought as mutant *KRAS* has been reported to upregulate SHH expression [37]. The majority of high Hh ligand expressing cell lines, defined as >4× expression of normal bronchial epithelial HBEC7-KT cells, also expressed mutant *KRAS* (Fig. 1c). H23, H2887, HCC44, and H2258 LAD cells expressed high levels of SHH protein, whereas H441 and H3122 expressed low levels of SHH protein as measured by immunoblot (Fig. 1d), consistent with qPCR results (Fig. 1c).

To test if SHH protein was secreted from LAD cells and could activate the Hh signaling pathway, we co-cultured three cell lines with the highest level of SHH (from Fig. 1c, d) with Hh-pathway responsive Shh-Light2 mouse embryonic fibroblasts that contain an 8×-GLI binding site-firefly luciferase reporter [38]. Treatment of Shh-Light2 cells alone with SHHN conditioned medium (CM) [39] induced high levels of Hh pathway activity that was suppressed by KAAD-cyclopamine 200 nM [40], a potent SMO antagonist, and 5E1 10 μg/ml, a blocking monoclonal antibody that binds to SHH and IHH [41, 42] (Fig. 1e). Co-culture of high SHH-expressing cells (H2887, H23, and HCC44) with Shh-Light2 cells, but without addition of exogenous SHH, resulted in potent activation of the pathway in Shh-Light2 cells, compared with normal airway epithelial HBEC7-KT cells (Fig. 1e). Treatment of these co-cultured cells with KAAD-cyclopamine and 5E1 inhibited Hh pathway activation in Shh-Light2 cells (Fig. 1e). In contrast, low SHH-expressing H3122 cells did not significantly induce Hh pathway activation in Shh-Light2 cells. To test for autocrine activation of the Hh signaling pathway in tumor cells, we treated high SHH-expressing H2887 and HCC44 cells (Fig. 1c, d) with recombinant SHH (rSHH) 1 μg/ml, 5E1 10 μg/ml or KAAD-cyclopamine 300 nM and monitored the mRNA transcription of reported pathway target genes *GLI1, PTCH1, HHIP, BMP4, BMP7, MYCN, CCND1, SOX9*, and *BMI1* by qPCR after treatment. In both H2887 (Fig. 1f–h, Supplementary Fig. 2) and HCC44 (Supplementary Fig. 3), addition of rSHH did not increase mRNA transcription of target genes nor did treatment with 5E1 or KAAD-cyclopamine substantially decrease mRNA transcription, defined as >50% decrease, across the panel of the tested target genes compared with DMSO control. These results are in contrast with Hh-responsive MLg murine lung fibroblasts [35] (Supplementary Fig. 4) suggesting that the tumor cells did not respond to secreted SHH in an autocrine manner. Interestingly, H2887 and HCC44 cells expressed higher *GLI1* mRNA and other pathway target genes than the normal bronchial epithelial HBEC7-KT cells (Fig. 1f–h, Supplementary Fig. 2) suggesting that the genes may be upregulated by a Hh-independent mechanism. Taken together, the results of the co-culture (Fig. 1e) and autocrine (Fig. 1f–h, Supplementary Figs. 2 and 3) experiments suggested that SHH from LAD cells activate the pathway in stromal cells in a paracrine manner without autocrine activation in tumor cells.

### SHH does not affect lung adenocarcinoma growth in vivo

**Fig. 2:**
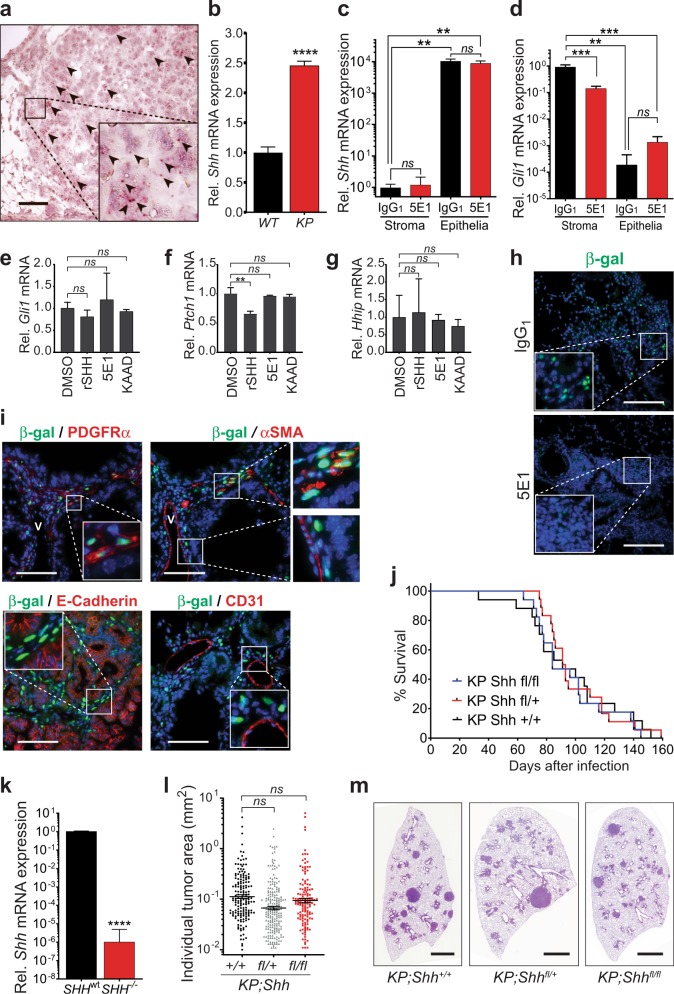
SHH does not affect tumor growth and survival in vivo. **a** *Shh* mRNA expression is shown in lung tumor tissues generated in *K-ras*^*G12D/+*^*;p53*^*fl/fl*^ (*KP*) mouse by RNA in situ hybridization. Red puncta indicate *Shh* mRNA. Inset shows an enlarged region for better clarity. Scale bar is 50 µm. **b** EpCAM+; GFP+ lung epithelial cells of *KPmTmG* mice 3 weeks after adeno-cre recombinase (adeno-cre) infection (‘KP’ in panel) and CD31−, CD45−, EpCAM+ lung epithelial cells of uninfected *KP* mice (‘Wt’ in panel) were FACS-sorted and *Shh* mRNA expression was analyzed by qPCR. The data represent mean of duplicates ± s.e.m (*n* = 4 lung lobes from 2 mice) for adeno-cre infected *KPmTmG* mice and mean of triplicates ± s.e.m (*n* = 4 lung lobes from 4 mice) for uninfected *KP* mice. ****P < 0.0001. **c, d** CD31−, CD45−, EpCAM+, GFP+ lung epithelial cells and CD31−, CD45−, EpCAM−, GFP− lung stromal cells of *KPmTmG* mice treated with 5E1 or IgG_1_ for 4 weeks starting 2 weeks after adeno-cre infection were FACS-sorted and (**c**) *Shh* and (**d**) *Gli1* mRNA expression were analyzed by qPCR. The data represent mean of triplicates ± s.e.m (*n* = 2 whole lungs from 2 mice per treatment arm). **P < 0.01, ***P < 0.001. *ns* not significant. **e-g** *KP* lung adenocarcinoma 808-T3 cells were treated with rSHH 1 μg/ml, 5E1 antibody 10 μg/ml, or KAAD-cyclopamine 300 nM and then mRNA expression of (**e**) Gli1, (**f**) *Ptch1*, (**g**) *Hhip* were measured by qPCR. The data represent mean of triplicates ± s.d.**P < 0.01. *ns* not significant. **h** Lung sections from *KP;Gli1*^*LacZ/+*^ mouse treated with 5E1 or IgG_1_ for 2 weeks starting 2 weeks after adeno-cre infection were stained for β-galactosidase (green) that represents GLI1. Insets show enlargement of boxed areas for better clarity. Scale bars are 50 µm. **i** Lung sections from *KP;Gli1*^*LacZ/+*^ mouse (4 weeks after adeno-cre infection) were co-stained for β-galactosidase (green) and PDGFRα, αSMA, E-Cadherin, or CD31 (Red). Tissue sections used for PDGFRα and αSMA stains are ~20 µm apart from each other. Insets show enlargement of boxed areas for better clarity. Scale bars are 50 µm. **j** Survival curves of *KP;Shh*^*+/+*^, *KP;Shh*^*fl/+*^, and *KP;Shh*^*fl/fl*^ mice after infection with adeno-cre are shown. *KP;Shh*^*+/+*^ *n* = 17, *KP;Shh*^*fl/+*^ *n* = 18, *KP;Shh*^*fl/fl*^ *n* = 17. **k** EpCAM + ;GFP + lung epithelial cells of *KPmTmG;Shh*^*WT*^ and *KPmTmG;Shh*^*fl/fl*^ mice 3 weeks after adeno-cre infection were FACS sorted and *Shh* mRNA levels were measured by qPCR. The data represent mean of duplicates + /− s.e.m. *n* = 4 lung lobes from 2 mice per treatment arm. The expression levels were normalized to *Shh*^*WT*^. ****P < 0.0001. **l** Quantification of individual tumor area is shown from mice 10 weeks after adeno-cre infection. Data represent mean of *KP;Shh*^*+/+*^ *n* = 162, *KP;Shh*^*fl/+*^ *n* = 206, *KP;Shh*^*fl/fl*^ *n* = 137 tumor ± s.e.m. **m** H&E images of left lung from *KP;Shh*^*+/+*^, *KP;Shh*^*fl/+*^, and *KP;Shh*^*fl/fl*^ mice 10 weeks after adeno-cre infection from **l**. Scale bars are 2 µm.

We next sought to test the role of stromal Hh pathway in lung tumor development. As reliable anti-SHH antibodies for immunohistochemistry (IHC) were not commercially available, we tested for *Shh* mRNA expression by in situ hybridization. We validated *Shh* mRNA probes in the neural tube of E11.5 mouse embryos, where SHH is highly expressed in the notochord [43, 44] and floor plate [45, 46] (Supplementary Fig. 5a). Ten weeks after infection of *Kras*^*Lox-Stop-Lox-G12D/*+^*;Trp53*^*fl/fl*^ (*KP*) mice [47] with adenovirus-expressing cre recombinase (adeno-cre) by intranasal inhalation, LAD expressed *Shh* mRNA as shown by in situ hybridization (Fig. 2a, Supplementary Fig. 5b). We further verified the expression of *Shh* mRNA specifically in primary *KP* transformed lung epithelia. Lungs from uninfected *KP* mice and *KP;Rosa26*^*Loxp-mtdTomato-Stop-Loxp-mGFP/+*^ (*KPmTmG*) mice [48], a strain that conditionally switches from constitutive tdTomato expression to GFP expression and initiates LAD when exposed to cre recombinase (Supplementary Fig. 6a), infected with adeno-cre were enzymatically dissociated into single cells. Lung epithelial cells were isolated using FACS–EpCAM+, GFP+ (adeno-cre infected cells) for *KPmTmG* epithelia (Supplementary Fig. 6b), and CD31− (endothelial cell antigen), CD45− (leukocyte antigen), EpCAM+ (epithelial cell antigen) for uninfected *KP* epithelia (Supplementary Fig. 6c)—and *Shh* mRNA measured by qPCR. Infected *KPmTmG* lung epithelia expressed higher levels of *Shh* mRNA than wild-type lung epithelia of uninfected *KP* mice (Fig. 2b). After identifying the optimal dose of 5E1 for in vivo studies using a Hh-dependent hair regrowth study [49, 50] (Supplementary Fig. 7), *KPmTmG* mice were treated with IgG_1_ control or 5E1 10 mg/kg by intraperitoneal (i.p.) injection twice per week for four weeks starting 2 weeks after adeno-cre infection and transformed epithelial cells and stromal cells were isolated by FACS (Supplementary Fig. 8). *Shh* mRNA expression was ~4 orders of magnitude higher in transformed lung epithelial cells than in stromal cells, as measured by qPCR (Fig. 2c). *Gli1* mRNA levels, as a measure of response to SHH ligand, were ~4 orders of magnitude higher in stromal cells than in transformed epithelial cells (Fig. 2d). Furthermore, stromal cells from *KPmTmG* mice treated with 5E1 showed ~90% decrease in *Gli1* mRNA transcription compared with stromal cells treated with IgG_1_ control in contrast to FACS-sorted epithelial cells (Fig. 2d), suggesting that the Hh signaling pathway is activated primarily in stroma by a paracrine mechanism with no autocrine activation in tumor epithelia. mRNA expression of Hh pathway target genes in 808-T3 cells, a murine *KP* LAD cell line that expresses SHH (Supplementary Fig. 9), were not significantly increased when treated with rSHH or significantly diminished when treated with pathway inhibitors, 5E1 or KAAD-cyclopamine (Fig. 2e–g, Supplementary Fig. 10). To identify the pathway-responsive stromal cells, we crossed *KP* mice with the *Gli1*^*Lacz/+*^ reporter strain [51] that contains the *LacZ* gene with a nuclear localization signal sequence knocked into the *Gli1* locus to generate the *KP;Gli1*^*Lacz/+*^ strain. Nuclear expression of β-galactosidase was diminished in *KP;Gli1*^*Lacz/+*^ murine lungs treated with 5E1 10 μg/ml twice per week for 2 weeks starting 2 weeks after adeno-cre infection compared with those treated with IgG_1_ control (Fig. 2h, Supplementary Fig. 11). Nuclear β-galactosidase co-localized with fibroblast (PDGRα) and myobfibroblast (αSMA) markers but not with perivascular smooth muscle (αSMA+, PDGRα−), lung epithelial cells (E-Cadherin+), nor endothelial cells (CD31+) (Fig. 2i, Supplementary Fig. 12) suggesting that fibroblasts and myofibroblasts are the primary cells that respond to Hh ligands. We next tested the requirement of stromal Hh pathway activity for LAD tumorigenesis by crossbreeding *KP* with *Shh*^*fl/fl*^ [52] mice to generate *KP, KP;Shh*^*fl/+*^, and *KP;Shh*^*fl/fl*^ strains to induce LAD with wild-type (wt), heterozygous, and homozygous loss of SHH expression. Surprisingly, *KP, KP;Shh*^*fl/+*^, and *KP;Shh*^*fl/fl*^ mice did not show any differences in survival after LAD induction with adeno-cre (Fig. 2j). To verify that *Shh* was indeed deleted in *KP;Shh*^*fl/fl*^ mice, we isolated EpCAM+;GFP+ infected lung epithelial cells by FACS from *KPmTmG* and *KPmTmG;Shh*^*fl/fl*^ mice 3 weeks after adeno-cre infection, analogous to Supplementary Fig. 6b, and tested for *Shh* mRNA expression by qPCR. Indeed, *KP;Shh*^*fl/fl*^ infected epithelial cells expressed *Shh* mRNA ~6 orders of magnitude less than *KP* epithelial cells (Fig. 2k), suggesting that *Shh* was indeed knocked out. Furthermore, no significant differences in tumor size were seen in the lungs of *KP, KP;Shh*^*fl/+*^, and *KP;Shh*^*fl/fl*^ 10 weeks after infection (Fig. 2l, m). Taken together, these results suggest that SHH may not play a role in mutant Kras LAD tumorigenesis and progression.

### Activation of the Hh pathway in stroma prolongs survival by restraining tumor growth and metastasis in vivo

**Fig. 3:**
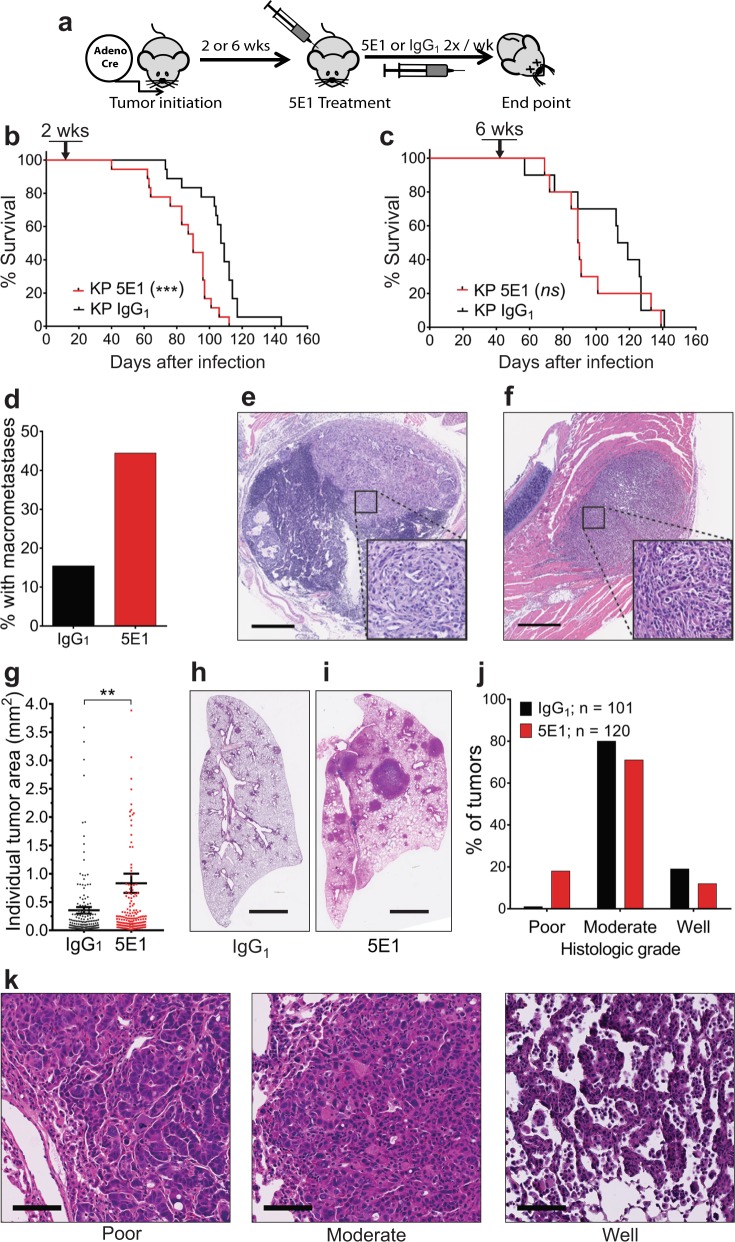
Inhibition of stromal Hh pathway activation worsens survival with increased tumor growth and metastases. **a** Schematic diagram of *KP* mice infection and treatment with 5E1 or IgG_1._ **b**, **c** Survival curves are shown of *KP* mice treated with 5E1 10 mg/kg twice per week or corresponding IgG_1_ dose starting, (**b**) 2 weeks (5E1 *n* = 18, IgG_1_ *n* = 18, ***P = 0.0002) and **c** 6 weeks (5E1 *n* = 10, IgG_1_ *n* = 10, *P* = 0.34, *ns* not significant) after tumor initiation by intranasal administration of adeno-cre. **d** Fraction of mice with grossly visible metastases from experiment in **b** is shown. **e**, **f** Representative H&E images of metastatic tumors in (**e**) a mediastinal lymph node and in (**f**) pleura invading the chest wall. Scale bars are 500 µm. **g**–**j** *KP* mice were treated with 5E1 10 mg/kg twice per week or corresponding IgG_1_ dose for 8 weeks starting 2 weeks after adeno-cre infection. **g** Quantification of individual tumor area is shown. Data represent mean of IgG_1_ (*n* = 195 tumors) or 5E1 (*n* = 182 tumors) ± s.e.m. **P < 0.01. **h**, **i** H&E images of left lung of mice from **g**. Scale bars in panels h and i are 2 and 2.5 mm, respectively. **j** Percent of tumors with poor, moderate, and well-differentiated histologies are shown of *KP* LAD 10 weeks after adeno-cre infection. Data represent mean of IgG_1_ (*n* = 101 tumors) or 5E1 (*n* = 120 tumors). **k** Representative H&E images of poor, moderate, and well differentiated tumors are shown. Scale bars are 100 µm.

To further examine the effect of paracrine Hh pathway activity in lung tumorigenesis, *KP* mice were treated with 5E1 10 mg/kg i.p. twice per week or IgG_1_ control starting 2 or 6 weeks after tumor initiation by adeno-cre infection (Fig. 3a) such that the pathway was inhibited early in the tumorigenic process (2 weeks) or once adenomas with nuclear atypia had been established (6 weeks) [47]. *KP* mice treated with 5E1 starting 2 weeks after tumor initiation had significantly worse survival compared with IgG_1_ treated control mice (Fig. 3b) in contrast to mice treated with 5E1 starting 6 weeks after adeno-cre infection (Fig. 3c). Furthermore, *KP* mice treated with 5E1 at the 2 week time point exhibited significantly higher rates of metastases (Fig. 3d), primarily to mediastinal lymph nodes and pleura (Fig. 3e, f). Examination of LAD tumors after 8 weeks of 5E1 treatment (10 weeks after adeno-cre infection) demonstrated significantly larger size of tumors (Fig. 3g–i) with a greater proportion of poorly differentiated tumors and less well-differentiated tumors (Fig. 3j, k) compared with mice treated with IgG_1_ control. Thus, pharmacologic inhibition of stromal Hh pathway induced greater tumor burden with greater metastases and worse survival, suggesting that stromal Hh pathway activity restrains LAD growth and metastasis.

### IHH is the predominant Hh ligand in murine mutant Kras lung adenocarcinoma

**Fig. 4:**
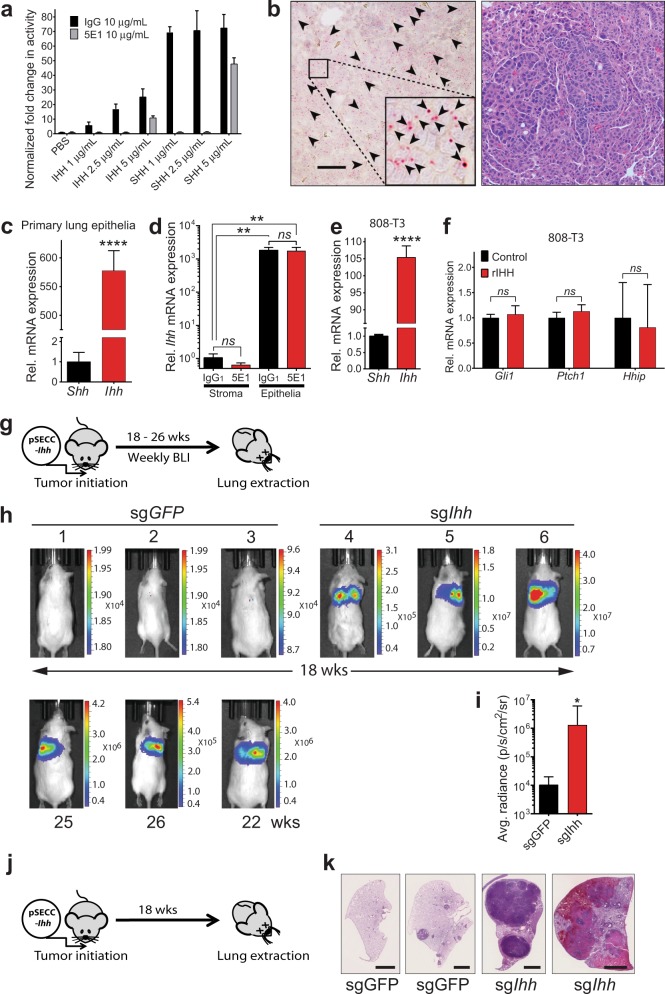
*IHH* regulates the suppression of lung adenocarcinoma. **a** Hh pathway activity, as measured by 8×-GLI-luciferase reporter relative to PBS control in Shh-Light2 reporter fibroblasts, is shown. Shh-Light2 cells were treated with 1, 2.5, and 5 µg/ml of mouse rIHH or rSHH in combination with 5E1 or IgG_1_ 10 µg/ml. PBS was used as a control vehicle for IHH or SHH. **b** In situ hybridization for *Ihh* mRNA (left panel) and corresponding H&E image (right panel) in LAD of *KP* mice are shown. Arrowheads point to regions of red puncta that indicate *Ihh* mRNA. Inset shows an enlarged region for better clarity. In situ hybridization and H&E images are ~65 µm apart. Scale bar is 50 µm. **c** *Shh* and *Ihh* mRNA levels of FACS sorted lung epithelial cells from *KPmTmG* mice 6 weeks after adeno-cre infection is shown. mRNA expression was measured by qPCR. Data represent mean of duplicate ± s.e.m. *n* = 4 mice. ****P < 0.0001 **d** CD31−, CD45−, EpCAM+, GFP+ lung epithelial cells and CD31−, CD45−, EpCAM−, GFP−, tdTomato+ lung stromal cells from *KPmTmG* mice treated with 5E1 or IgG_1_ for 4 weeks starting *2* weeks after adeno-cre infection were FACS-sorted and then *Ihh* mRNA expression was analyzed by qPCR. The data represent mean of triplicates ± s.e.m. *n* = 2 mice per treatment arm. **P < 0.01. *ns* not significant. **e** *Shh* and *Ihh* mRNA levels from 808-T3 cells are shown as measured by qPCR. Data represent mean of triplicates ± s.e.m. ****P < 0.0001. **f** 808-T3 cells were treated with rIHH 2.5 μg/ml or control vehicle and expression of *Gli1*, *Ptch1*, and *Hhip* mRNA were measured by qPCR. The data represent mean of triplicates ± s.d. *ns* = not significant. **g** Schematic diagram of the experiment for **h** and **i**. *KP;Rosa26*^*LSL-fLuc/+*^ mice were infected with 5 × 10^4^ ifu pSECC-*Ihh* or pSECC-*GFP* and tumor growth monitored by bioluminescence (*n* = 3 for each treatment arm). **h** Bioluminescence images (BLI) are shown of lung tumors in *KP;Rosa26*^*LSL-fLuc/+*^ 18 weeks after infection with lentiviral pSECC-sg*Ihh or* pSECC-sg*GFP* in the first row. pSECC-sg*GFP* mice were continued to be monitored and the second row shows BLI of mice 22–26 weeks after pSECC-sg*GFP* infection. **i** Quantification of luminescence intensity is shown for pSECC-sg*Ihh* and pSECC-sg*GFP* infected mice at 18 weeks (*n* = 3 per treatment arm). *P < 0.05. **j** Schematic diagram of the experiment for **k**. *KP;Rosa26*^*LSL-fLuc/+*^ mice were infected with 5 × 10^4^ ifu pSECC-*Ihh* or pSECC-*GFP* and lungs obtained at 18 weeks after infection (*n* = 2 mice per treatment arm). **k** Representative H&E images of right upper lobes from pSECC-sg*Ihh* and pSECC-sgGFP infected mice at 18 weeks. Scale bars are 2 mm.

With the disparate outcomes of genetic SHH loss (Fig. 2j, l, m) and pharmacologic blockade by 5E1 (Fig. 3b, d–k), we hypothesized that IHH may play a role in LAD tumorigenesis as 5E1 binds both SHH and IHH. We verified that 5E1 can inhibit stromal pathway activation by IHH using Shh-Light2 cells stimulated with either recombinant IHH (rIHH) or rSHH (Fig. 4a). Of note, there was almost no induction of pathway activity with recombinant DHH treatment (results are not shown). As reliable antibodies for IHH IHC and immunoblots were not commercially available, we turned to RNA in situ hybridization. *KP* LAD 10 weeks after adeno-cre infection expressed *Ihh* mRNA (Fig. 4b). To further verify that IHH is expressed by transformed lung epithelial cells, EpCAM+,GFP+ epithelial cells were isolated by FACS (analogous to Supplementary Fig. 6b) from *KPmTmG* mice 6 weeks after adeno-cre infection. The sorted epithelial cells show a striking increase in *Ihh* mRNA expression compared with *Shh* mRNA as measured by qPCR (Fig. 4c). In FACS-sorted lung epithelial and stromal cells (analogous to Supplementary Fig. 8), *Ihh* mRNA was expressed primarily in lung epithelial cells (Fig. 4d) with stromal cells responding to Hh ligands (Fig. 2d). *Ihh* mRNA was also expressed significantly higher than *Shh* mRNA in 808-T3 murine *KP* LAD cell line (Fig. 4e). Addition of more rIHH to 808-T3 cells did not modulate transcription of pathway target genes (Fig. 4f, Supplementary Fig. 13) in contrast to MLg lung fibroblasts (Supplementary Fig. 14), further suggesting that there is no autocrine activation of the Hh signaling pathway in tumor cells. To genetically test the requirement of IHH to suppress LAD tumorigenesis and growth, we used the pSECC lentiviral in vivo CRISPR/Cas9 system [53] that encodes for Cre recombinase to initiate tumorigenesis, Cas9 for gene editing, and sgRNA against the gene of interest. Several candidate sgRNA against *Ihh* (sg*Ihh*) were tested with SURVEYOR assay (Supplementary Fig. 15a) and the sgRNA sequence (#2, hereafter just sg*Ihh*) with the greatest percentage of digested bands was chosen for further study. We tested pSECC-*Ihh* for loss of *Ihh* mRNA expression by qPCR in 808-T3 cells with high *Ihh* mRNA expression (Fig. 4e). Approximately half of the clones from 808-T3 cell lines transfected with the pSECC-*Ihh* showed substantial decreases in *Ihh* mRNA expression compared with pSECC-*GFP* control (Supplementary Fig. 15b). Subsequently, *KP;Rosa26*^*LSL-fLuc/+*^ (*KPL)* mice were infected with lentiviral particles containing pSECC-*Ihh* or pSECC-*GFP* via intratracheal administration and tumor growth monitored by bioluminescence imaging (BLI) (Fig. 4g). Infection with pSECC-*Ihh* induced significant tumor growth compared with pSECC-*GFP* control 18 weeks after infection (Fig. 4h, i). *KPL* mice infected with pSECC-*GFP* eventually developed tumors that were detected by BLI at 22–26 weeks after infection (Fig. 4h). Examination of tumors at 18 weeks after pSECC-*Ihh* or pSECC-*GFP* infection from a separate experiment demonstrated greater tumor burden with loss of IHH (Fig. 4j, k).

### IHH in human lung adenocarcinoma

**Fig. 5:**
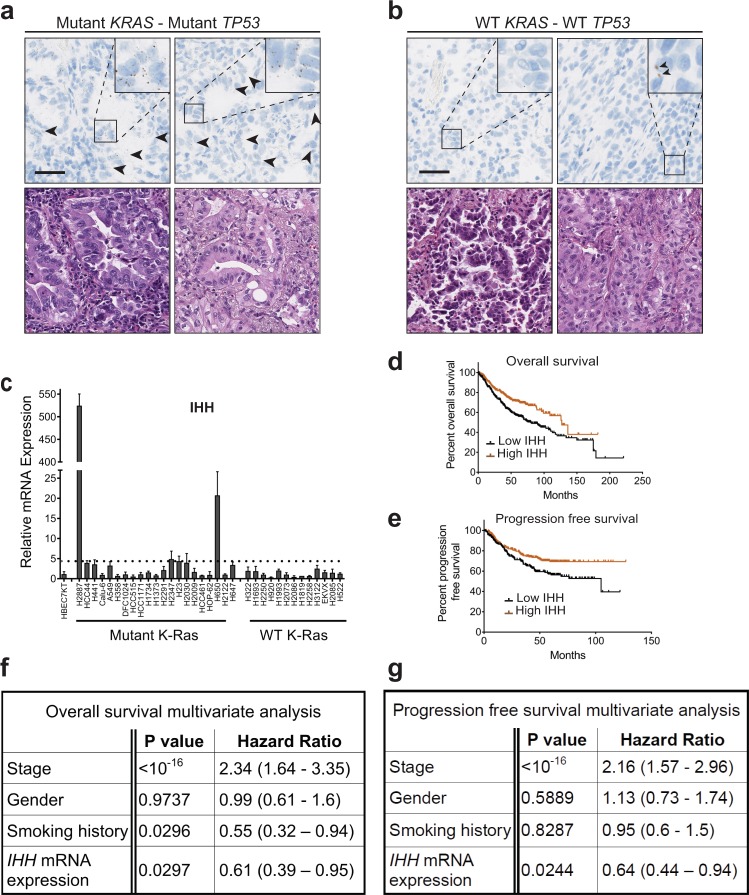
IHH in human lung adenocarcinoma. **a**, **b** In situ hybridization for *IHH* mRNA (top panels) in (**a**) mutant *KRAS*—mutant *TP53* and (**b**) wild-type WT *KRAS* and WT *TP53* human LAD are shown. Brown puncta indicate *IHH* mRNA. Arrowheads indicate regions of *IHH* mRNA staining in malignant cells. Insets show enlarged regions for better clarity. H&E images of tumors corresponding to ISH images above are shown in the bottom panels. Scale bar is 50 µm. **c** Expression of *IHH* mRNA as measured by qPCR relative to a normal bronchial epithelial cell line (HBEC7-KT). Dashed line represents 4× expression relative to HBEC7-KT. **d**–**g** Survival analyses of lung adenocarcinoma patients with high and low *IHH* mRNA expression from Kaplan–Meier Plotter database [36]. *n* = 673 patients. High and low mRNA expression is relative to median expression. Kaplan-Meier plots by univariate analysis of (**d**) overall survival (*P* = 0. 0001) and (**e**) progression-free survival (*P* = 0.0069) of LAD patients are shown. Multivariate analysis of (**f**) overall survival and (**g**) progression-free survival of LAD patients is shown with stage, gender, smoking history, and *Ihh* mRNA expression as variables.

We next tested for *IHH* mRNA by in situ hybridization in human LAD samples in mutant and wild-type *KRAS* and *TP53* samples. Two of the three mutant *KRAS;TP53* samples expressed *IHH* mRNA in malignant cells (Fig. 5a, Supplementary Fig. 16), whereas only one of the six wild-type samples expressed *IHH* mRNA (Fig. 5b, Supplementary Fig. 16). All of the *IHH* mRNA positive tumors had a predominance of lepidic histology with mucinous features (Supplementary Fig. 16). Lepidic histology has been correlated with less aggressive biology. The prognosis of mucinous histology in LAD is uncertain currently [54]. Re-examination of the 34 human LAD cell lines (Fig. 1c) revealed only 4 lines with *IHH* mRNA elevated beyond four times the normal lung epithelial line HBEC7-KT (Fig. 5c). As most of the cell lines were generated from patients with late stage or metastatic adenocarcinomas, the dearth of cancer lines with upregulated *IHH* mRNA corroborates the in situ results of *IHH* mRNA in more indolent lepidic histologies (Fig. 5a, Supplementary Fig. 16). In high *IHH* mRNA expressing H650 cells (Fig. 5c), treatment with rIHH or pathway inhibitors, 5E1 and KAAD-cyclopamine, did not show increase nor substantial decrease (>50%) in mRNA transcription compared with DMSO control across the panel of tested pathway target genes, respectively (Supplementary Fig. 17). These data, along with those of high *SHH/IHH* expressing H2887 cells (Fig. 1f–h, Supplementary Fig. 2) suggest that there is no autocrine activation of the pathway by IHH in human LAD cells. Univariate Cox regression analysis of a clinically annotated microarray database of human LAD (KM Plotter; [36]) revealed that patients with high expression of *Ihh* mRNA had better overall (*P* = 0.0001; Fig. 5d) and progression-free (*P* = 0.0069; Fig. 5e) survival compared with those with low expression. These results remained consistent after multivariate analyses when stage, gender, and smoking history were considered (Fig. 5f, g), in agreement with our murine LAD data (Figs. 3b and 4g–k). The data here suggest that IHH is sufficient to suppress tumor initiation and growth and that SHH is dispensable for LAD tumorigenesis.

### Loss of stromal Hh pathway inhibits angiogenesis and increases the activity of reactive oxygen species

**Fig. 6:**
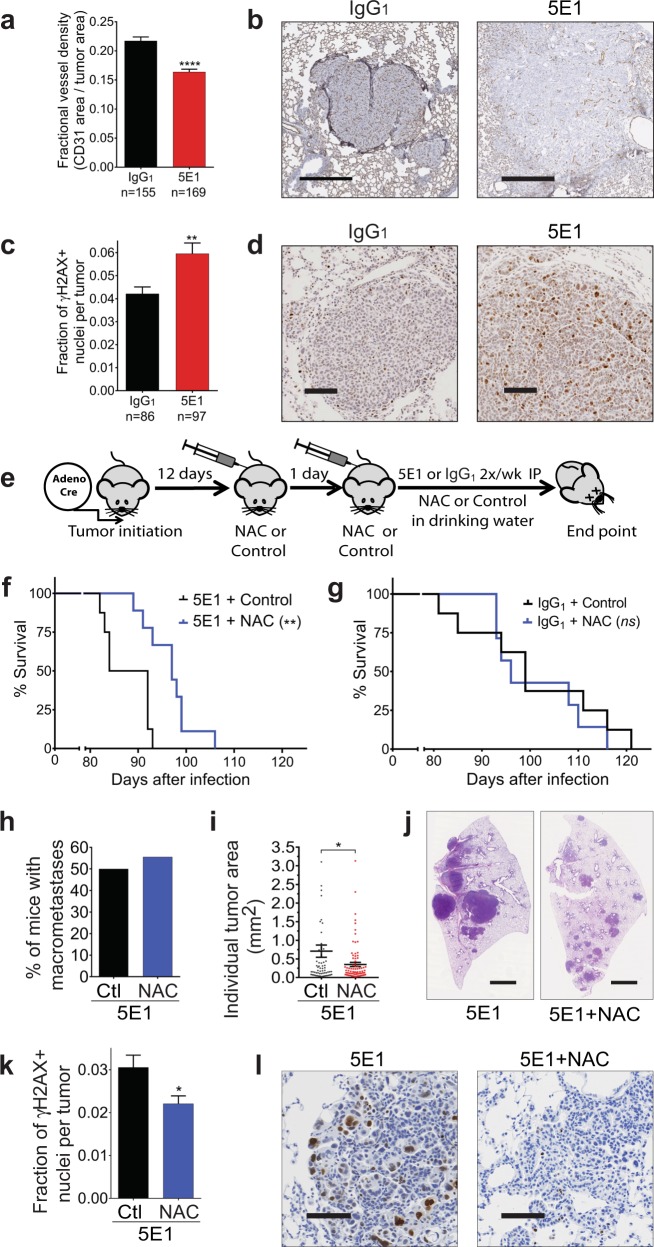
*IHH* loss inhibits angiogenesis and increases activity of reactive oxygen species. **a**–**d** *KP* mice were treated with 5E1 10 mg/kg twice per week or corresponding IgG_1_ dose for 8 weeks starting 2 weeks after infection **a** Quantification of vessel density (area of CD31 positive cells in tumor/tumor area) is shown. Data represent mean of IgG_1_ (*n* = 155 tumors) or 5E1 (*n* = 169 tumors) ± s.e.m. ****P < 0.0001. **b** Images of lung tumors of *KP* mice stained for CD31 by IHC with DAB substrate. Scale bar is 500 µm. **c** Fraction of γH2AX+ nuclei (γH2AX+ nuceli per tumor/total nuclei per tumor) is shown. Data represent the mean of IgG_1_ (*n* = 86 tumors) or 5E1 (*n* = 97 tumors) ± s.e.m. **P < 0.01 **d** Images of lung tumors of *KP* mice stained for γH2AX by IHC. DAB was used as substrate. Scale bar is 100 µm. **e** Schematic diagram of survival study for **f**–**h**. *KP* mice were infected with adeno-cre by intranasal inhalation and treated with vehicle or *N*-acetyl cysteine (NAC) 200 mg/kg i.p. once per day on days 12 and 13 after adeno-cre infection. From day 14, mice were treated with 5E1 10 mg/kg i.p. twice per week or corresponding IgG_1_ dose and NAC 1 g/L supplemented in their drinking water. **f** Survival curves are shown of *KP* mice treated with 5E1 and control vehicle (*n* = 8) or NAC (*n* = 9). **P = 0.0031. **g** Survival curves of *KP* mice treated with IgG_1_ with control vehicle (*n* = 8) or NAC (*n* = 7, *P* = 0.55) starting 2 weeks after infection. *ns* not significant. **h** Fraction of mice with grossly visible metastases from experiment in **f** is shown. **i** Quantification of individual lung tumor area of *KP* mice treated with 5E1 in combination with control or NAC for 8 weeks starting 2 weeks after adeno-cre infection. Data represent the mean of Ctl (*n* = 64 tumors) and NAC (*n* = 85 tumors) ± s.e.m. *P < 0.05. **j** H&E images of left lung from **i**. Scale bars are 2 mm. **k** Fraction of γH2AX+ nuclei of tumors is shown. Data represent the mean of Ctl (*n* = 87 tumors) and NAC (*n* = 82 tumors) ± s.e.m. *P < 0.05. **l** Images of lung tumors of *KP* mice from (**k**) stained for γH2AX by IHC. DAB was used as substrate. Scale bars are 100 µm.

The Hh signaling pathway has been implicated in the regulation of angiogenesis in normal tissues [55, 56] and cancer [57, 58] through induction of angiogenic factors including VEGFs and ANG1, 2. Examination of CD31 expression, a marker of endothelial cells, showed decreased blood vessel density in LAD tumors treated with anti-SHH/IHH 5E1 antibody compared with IgG_1_ treated tumors (Fig. 6a, b). As the effects of stromal Hh pathway inhibition were seen with mice when treatment was initiated 2 weeks after adeno-cre infection (Fig. 3b, d–k), we hypothesized that the inability of growing tumors to generate new vessels would lead to early hypoxia and production of reactive oxygen species (ROS) [59, 60], that in turn, would promote tumor proliferation and growth [61–63]. We developed two macros (“*ROI_Draw”* and “*Nuclear_Fraction_Calculator”*) for ImageJ [64] or Fiji [65] to quantify DAB stained nuclei of phospho-histone 2AX (γH2AX), a protein that responds to double stranded DNA breaks and a marker of oxidative stress [66, 67]. *Nuclear_Fraction_Calculator* counts DAB stained nuclei and total nuclei in digital images of tissue sections and calculates the fraction of IHC positive nuclei within regions of interest (ROI; tumors in our studies) that have been drawn interactively with *ROI_Draw*. With these macros, LAD from mice treated with 5E1 showed significantly higher fraction of nuclei stained with γH2AX than tumors from IgG_1_ treated mice (Fig. 6c, d), suggesting increased DNA damage from ROS. To assess whether ROS from stromal Hh pathway inhibition induced accelerated tumor growth, *KP* mice were treated with 5E1 and *N-*acetyl cysteine (NAC), as a scavenger of ROS and precursor to the antioxidant, glutathione (GSH) (Fig. 6e). Treatment with NAC and 5E1 prolonged survival compared with 5E1 and vehicle control (Fig. 6f), whereas treatment with NAC and IgG_1_ did not affect survival (Fig. 6g). Furthermore, the median survival of 5E1 with NAC approximated that of IgG_1_ with vehicle control (Supplementary Fig. 18). Interestingly, the rate of metastases did not decrease when mice were treated with 5E1 and NAC compared with 5E1 and vehicle control (Fig. 6h). The tumor size in mice treated with 5E1 and NAC were significantly decreased compared with mice treated with 5E1 and vehicle control 10 weeks after adeno-cre infection (Fig. 6i, j) and corresponded to decreased DNA damage as measured by γH2AX stained nuclei as a marker of ROS activity (Fig. 6k, l). These data suggest that IHH restrains tumor growth through support of angiogenesis and limiting ROS activity early in the tumorigenic process.

## Discussion

In accordance with previous studies [20–25], paracrine Hh activation of stroma, particularly early in the tumorigenic process, suppresses lung tumor growth, formation of aggressive histologies and metastases. A surprising result of our studies was the central role of IHH, instead of SHH, to suppress tumor growth. SHH is the dominant ligand that regulates lung development [33], adult lung airway homeostasis [34], and lung cancers [26, 27, 30, 32, 35]. IHH is expressed in the adult colon and prostate and restrains the growth of colon [24, 68] and prostate [25] cancers. However, to our knowledge, IHH activity has not been reported in the lung. Further studies are needed to test if IHH has a role in the homeostasis of the adult lung epithelia or if it is unique to lung cancers.

In our studies, loss of stromal pathway activation in *KP* LAD decreased blood vessel density (Fig. 6a, b) suggesting that the Hh signaling pathway induces angiogenesis in the lungs consistent with reports in other organs [55, 56, 69]. However, loss of stromal Hh pathway activation in *KP* pancreas ductal adenocarcinoma (PDAC) increased tumor blood vessel density and inhibition of angiogenesis through VEGFR2 antagonism in *KP;Shh*^*fl/fl*^ PDACs prolonged mouse survival [20]. Another study reported that loss of Hh ligand co-receptors, GAS1 and BOC, in mouse embryonic and pancreas cancer-associated fibroblasts (CAFs) led to partial suppression of pathway response to SHH and increased angiogenesis [70]. Loss of co-receptors GAS1, BOC, and CDO in fibroblasts caused a more severe suppression of the pathway and inhibited angiogenesis through modulation of angiogenic ligands VEGFA, ANGPT1, 2 [70]. If stromal cells respond distinctly to SHH and IHH ligands, then IHH may play a more prominent role in angiogenesis in LAD than SHH due to the lower potency of IHH (Fig. 4a) analogous to the diminished pathway response of *Gas1*^−/−^;*Boc*^−/−^ fibroblasts in pancreatic cancer [70]. Previous studies also have noted differences in genomic and transcriptomic heterogeneity [71] and effectors downstream of mutant Kras [72] between murine *KP* LADs and PDACs. Such differences may also play a role in the tumor microenvironment where responses to Hh ligands may differ significantly between pancreas and lung stroma. The distinct phenotypic outcomes of stromal Hh pathway activation in LAD and PDAC suggest that tumor-stromal interactions of various cancer types will need to be studied individually and caution against broad generalizations.

ROS exhibit seemingly paradoxical effects of tumor growth enhancement and tumor cytotoxicity depending on their levels [73]. Oncoproteins, such as mutant KRAS and MYC, and hypoxic states can increase cellular ROS levels [63, 74] that enhance tumor growth [63, 75–77]. But high levels of ROS can be cytotoxic and cancer cells upregulate antioxidant proteins including glutathione peroxidases, peroxiredoxins, and NRF2 to maintain ROS at optimal levels [74]. Here, we have shown that loss of stromal pathway activity early in the tumorigenic process increased DNA damage as marker of ROS activity in tumor cells (Fig. 6c, d). Reduction of ROS activity with NAC combined with stromal pathway inhibition prolonged survival with retardation of tumor growth in *KP* LAD (Fig. 6f, i–l). A recent study [78] demonstrated increased metastases when *KP* mice were treated with NAC. In our study, addition of NAC to 5E1 treatment did not change the rate of metastases in *KP* mice compared with 5E1 treatment (Fig. 6h) while 5E1 treatment of *KP* mice increased the rate of metastases compared with control treatment (Fig. 3d–f). These results suggest that *KP* mice treated with 5E1 and NAC may have increased metastases compared with *KP* control mice and are in general agreement with the observations of Wiel et al. [78]. Further studies will be needed for direct comparisons of adding NAC to control or 5E1 treatment in *KP* mice.

In bladder [23] and colon [24] cancers, BMPs secreted from Hh-dependent stroma limit the histologic progression of cancers. Similarly, loss of stromal Hh pathway activation in the lung leads to higher grade tumors (Fig. 3j, k) and murine lung fibroblasts express BMPs in response SHH (Supplementary Fig. 4) and IHH (Supplementary Fig. 14). Thus, loss of BMPs from lung fibroblasts may also contribute to the increased growth and aggressiveness of *KP* LAD with pathway inhibition.

Our studies here highlight the tumor suppressive roles of stromal Hh pathway activation by IHH via limiting hypoxia and ROS activity through angiogenesis and reinforce the anti-oncogenic role of stroma early in the tumorigenic process. Identification of factors that negatively regulate IHH production in LAD may serve as targets of small molecule or antibody therapeutics to enhance IHH expression and restrain tumor growth and metastases. Such therapeutic strategies may be employed in early stage or locally advanced disease prior to surgery/high dose radiation or concurrent chemoradiation, respectively, where treatment failure often occurs due to distant metastases. Also, identification of such factors may serve as biomarkers to determine the early stage patients that might benefit from more aggressive therapy.

## Materials and methods

### Cell culture

All human LAD cell lines were obtained from the Hamon Cancer Center Collection (UT Southwestern Medical Center, UTSW), were DNA fingerprinted with a PowerPlex 1.2 kit (Promega) and tested for mycoplasma using e-Myco kit (Boca Scientific). The cell lines were generated between 1979 and 2007. Cells were maintained in RPMI-1640 (Life Technologies) with 5% fetal bovine serum (FBS). 808-T3 and Green-Go [53] cell lines were kind gifts from Dr David McFadden (UTSW) and Dr Tyler Jacks (MIT), respectively, and were maintained in DMEM (Life Technologies) with 10% FBS. All cells were maintained at 37 °C, with 5% CO2, and under humidified conditions.

### Drugs and reagents

5E1 antibody was expanded in our laboratory (see Supplementary material and methods) and prepared in PBS. IgG_1_ (InVivoMab, BE0083) was diluted in PBS. KAAD-cyclopamine (Millipore) was prepared in DMSO. Recombinant SHH (C25II) (R&D Systems) and IHH (C28II) (Genscript) were prepared in PBS containing 0.1% bovine serum albumin (BSA). N-Acetyl-L-cysteine (NAC) was purchased from Sigma-Aldrich and prepared in PBS for i.p. injection or sterile tap water for supplemented drinking water. For NAC solution, pH was adjusted to 7.4.

### GLI-reporter assay

Shh-Light2 cells [38], a clonal NIH-3T3 cell line that stably expresses 8xGLI-binding site-firefly and TK-*Renilla* luciferase reporters, were co-cultured with LAD cell lines in 24-well plates until confluent and then treated with KAAD-cyclopamine (Millipore) 200 nM, 5E1 antibody 10 µg/ml or recombinant SHHN protein 1 µg/ml in DMEM containing 0.5% (vol/vol) bovine calf serum. Luciferase activity was measured by Fluostar Optima (BMG Labtech) using Dual Luciferase Assay Reporter System (Promega).

### Quantitative real-time PCR

Total RNA was extracted using TriZol (Invitrogen) and purified with PureLink RNA Mini Kit (Invitrogen). cDNA was generated using iScript cDNA synthesis kit (Bio-Rad) or Superscript III First Strand Synthesis System (Invitrogen). qPCR was performed using Bio-Rad CFX real-time cycler and SYBR Green Master Mix (Bio-Rad). Data are presented as fold change relative to control samples using the ΔΔCt (2^−ΔΔCt^) method with *HPRT1 or GAPDH* as an internal control gene. Primers for qPCR are listed in Supplementary Table 1.

### Western blot

Cell lysates were generated and analyzed as previously described [31]. Briefly, cells were lysed in ice-cold lysis buffer (M-PER Mammalian Protein Extraction Reagent (Thermo Scientific) with protease inhibitors (Roche) and PhosSTOP phosphatase inhibitors (Roche). Cell lysates were centrifuged at 14,000 rpm for 5 min at 4 °C and then supernatants were collected. Protein concentration was measured using BCA protein assay kit (Pierce) following the manufacturer’s instructions. The following primary antibodies were used: SHHN (1:1000, Cell Signaling Technology, C9C5), HSP90 (1:2000, Santa Cruz biotechnology, sc-13119), and Tubulin (1:5000, abcam, ab7291).

### sg-RNA design and cloning

All sg-RNA against *Ihh* were designed using GE Dharmacon web tool. The sg-RNA sequences targeting GFP were published previously [79]. sg-RNA oligo candidates (listed on Supplementary Table 2) were inserted into pSECC vector (a kind gift from Dr Tyler Jacks, Addgene, 60820) by following the protocol available at this website: https://tinyurl.com/y29utjk8.

### Co-transfection of 808-T3 cells

Cells were grown to 70% confluency on six-well plates and then co transfected with pCMV:DsRed(FRT)GFP plasmid expressing DsRed (Addgene, 31128) and pSECC-*Ihh* or pSECC-*GFP* using Lipofectamine 3000 (Thermo Fisher Scientific) following manufacturer instructions. DsRed+ transfected cells were FACS sorted and plated at limiting dilutions to isolate clonal lines.

### Animals

All animal related experiments and procedures were performed with prior approval of the Institutional Animal Care and Use Committee at UTSW. FVB, *Kras*^*Lox-Stop-Lox-G12D/*^+ [80]*, Trp53*^*fl/fl*^ [81]*, Shh*^*fl/fl*^ [52], and *Rosa26*^*Lox-mtdTomato-Stop-Lox-mGFP/+*^ [48] mouse strains were purchased from Jackson Laboratory (Bar Harbor, ME). *Gli1*^*LacZ/+*^ [51] mouse strain was a kind gift from Dr Philip Beachy (Stanford University). Compound strains were generated through cross-breeding. For all animal experiments, mice were randomly selected to the experimental groups. Sample size for time point and survival studies included at least five mice per treatment arm except where noted in the figure legends. Numbers of mice used in the studies are given in the corresponding figure legends. Investigators were not blinded to the treatment groups.

### Infection and treatment of mice

Adenovirus-expressing cre recombinase (Ad5-CMV-Cre) was purchased from Vector Development Laboratory (Baylor College of Medicine, Houston). Six-to-ten-week-old mice were infected by intranasal instillation with 3 × 10^8^ pfu per mouse as described previously [82] to initiate lung tumorigenesis. For the in vivo CRISPR experiments, 10–14-week-old *Kras*^*Lox-Stop-Lox-G12D/*^^*+*^*; Trp53*^*fl/fl*^*;Rosa26*^*LSL-fLuc/+*^ (*KPLuc*) mice were infected with 5 × 10^4^ ifu of lentivirus containing pSECC-*Ihh* or pSECC-GFP via intratracheal administration as described previously [82]. *KP* or *KP;Rosa26*^*Lox-mtdTomato-Stop-Lox-mGFP/+*^ *(KPmTmG)* mice were treated with 5E1 or IgG_1_ 10 mg/kg intraperitoneally (i.p.) twice per week starting 2 or 6 weeks after adeno-cre infection. For NAC study, *KP* mice were infected with adeno-cre then treated with NAC 200 mg/kg i.p. on days 12 and 13 after adeno-cre infection. Afterward, NAC 1 g/L (pH = 7.4) was provided in the drinking water. Supplemented drinking water was changed every 2–3 days for the duration of study.

### Lung tissue extraction and processing

Mice were anesthetized with Avertin 25 mg/kg i.p., lungs perfused with ice-cold PBS, inflated with ice-cold 4% Paraformaldehyde (PFA) in PBS by intra-tracheal instillation, then fixed in 4% PFA at 4 °C for 24 h. Tissue processing and paraffin embedding were performed by Tissue Management Core Facility or Histo-Pathology Core Facility at UTSW. Frozen lung tissue blocks were made by inflating lungs with 50% (v/v) OCT (Tissue-Tek) in PBS and embedded in cryomold with 100% OCT on dry ice, and stored in −80 °C. Five and fifteen micron thick sections were made from each PFA fixed paraffin-embedded and frozen tissue blocks, respectively, and subjected to hematoxylin and eosin (H&E) or IHC staining. Brightfield images were taken using a Nikon Eclipse E800 or Hamamatsu Nanozoomer in Whole Brain Microscopy Facility (UTSW). Tumor area on H&E stained images were measured using NIS Elements (Nikon) or Fiji imaging software. The fraction of IHC positive nuclei in each tumor was estimated using ImageJ or Fiji as described in Supplemental material and methods. Images of Immunofluorescence stained sections were taken by Nikon Eclipse TE2000-U.

### Immunohistochemistry (IHC)

Heat-mediated antigen retrieval (citrate buffer, pH 6) was used for tissue sections from paraffin-embedded blocks. Samples were blocked with goat (Sigma) or donkey serum (Sigma) for 1 h and diluted primary antibodies were applied overnight at 4 ^o^C. Vectastain ABC (Vector Labs) with DAB substrate (Vector Labs) was used for staining according to the manufacturer’s instructions. The following primary antibodies were used: Ser139-p-Histone H2A.X (1:1,000; Cell Signaling Technology, 9718), CD31 (1:500, Cell Signaling Technology, 77,699), β-Galactosidase (1:20,000, abcam, ab9361), PDGFRα (Cell Signaling Technology, 3174), αSMA (1:500, Bio Care Medical, CM001A), and E-Cadherin (1:400, Cell Signaling Technology, 3195).

### RNA in situ hybridization method (RNAScope)

#### **M**urine samples

Five micrometer sections from paraffin embedded lungs were deparaffinaized, fixed in 10% formalin solution at room temperature for 24 h and then subjected to RNAscope assay using RNAscope 2.0 HD Reagent Kit-Red (Advanced Cell Diagnostics (ACD, 310034) and following manufacturer instructions. Mm-Ihh-noXHs (413091) and Mm-Shh (314361) probes were used for murine *Ihh* and *Shh* mRNA detection, respectively. Dapb (negative control, 310043) and PPIB (positive control, 313911) were used for quality control (data not shown).

#### **Human Samples**

Use of human samples for research purposes was approved by the Institutional Review Board at M.D. Anderson Cancer Center. Consent was obtained from patients for use of their samples for research purposes. Please see Supplementary methods for full details of methods. Briefly, in situ hydbridization was performed on an automated Leica Bond RX autostainer (Leica Biosystems, Nussloch, GmbH). LS 2.5 Probe- Hs-IHH probe (472388, ACD) was used. RNA expression of IHH was scored using a semi-quantitative scoring system as follows: 0: no staining or <1 dot/10 cells; 1+: 1–3 dots/cell; 2+: 4–9 dots per cell, None or very few dot clusters; 3+: 10–15 dots/ cell and <10% dots are in clusters; 4+: >15 dots/cell and >10% dots are in clusters. Positive (PPIB, Hs-PPIB, 313908) and negative (Dapb, Hs-PPIB, 312038) control probes were also evaluated, dapB score of <1 and PPIB score ≥2 with relatively uniform PPIB signals throughout the sample were considered adequate for analysis (data not shown).

### Histology analysis

H&E stained lungs with tumors from *KP* mice were examined. The pathologist was blinded to the conditions of the experiment. As nearly all tumors <0.5 mm were well-differentiated histology, only tumors ≥0.5 mm were examined. Tumors were graded as poor, moderate or well differentiated cancers.

### Digestion of lung tissue and FACS-sort of lung epithelial cells

Single cell suspensions of whole lungs were prepared as described previously [83]. For FACS, single cell suspensions were incubated with eBioscience Fixable Viability Dye eFluor™ 780 (Invitrogen) and the following antibodies (0.6 μg per 10^**7**^ cells): PerCP-Cy5.5 Rat Anti-Mouse CD45 (BD Pharmingen, 550994), PE-Cy7 Rat Anti-Mouse CD31 (BD Pharmingen, 561410), and Brilliant Violet 421 anti-mouse CD326 (Ep-CAM) (BioLegend, 118225) on ice for 45 min, and then subjected to FACS-sorting using FACS Aria II (BD Biosciences) at the Moody Foundation Flow Cytometry Core Facility at the Children’s Research Institute at UTSW. Flow cytometry data were analyzed with FlowJo v10.

### Statistical analysis

GraphPad Prism 7 software was used to generate the graphs and for statistical analysis. Unpaired, two-sided Student’s *t*-test was used for comparison of 2 groups. Mantel-Cox log-rank test was used for statistical significance of murine survival curves. Univariate Cox regression analysis was performed to calculate hazard ratio and log-rank *P* values per KM-Plotter [36] (http://kmplot.com/analysis/) for the human LAD Kaplan–Meier curves.

## Supplementary information

Supplemental Information

ROI_Draw

Nuclear_Fraction_Calculator
