# Supplementary material for: Stromal Hedgehog pathway activation by IHH suppresses lung adenocarcinoma growth and metastasis by limiting reactive oxygen species
Source: Oncogene. 2020 Feb 27;39(16):3258–75. doi: 10.1038/s41388-020-1224-5 (PMC7160060; doi:10.1038/s41388-020-1224-5)
Supplement: Supplementary file 1 — Supplemental Information [file 41388_2020_1224_MOESM1_ESM.pdf]

Supplemental Information

Stromal Hedgehog pathway activation by IHH suppresses lung adenocarcinoma growth and metastasis by limiting reactive oxygen species

Sahba Kasiri, Baozhi Chen, Alexandra N. Wilson, Annika Reczek, Simbarashe Mazambani, Jashkaran Gadhvi, Evan Noel, Ummay Marriam, Barbara Mino, Wei Lu, Luc Girard, Luisa M. Solis, Katherine Luby-Phelps, Justin Bishop, Jung-Whan Kim, and James Kim

<u>**Inventory of Supplemental Information**</u>

- **Supplemental Data, Methods, and References**
  - Supplement Fig. 1-18
  - Supplement Table 1- qPCR primers
  - Supplement Table 2 -Murine sgRNA sequences
  - Supplement Material and Methods
  - Supplement References
- **Custom Macros for ImageJ/Fiji**
  - *ROI_Draw*
  - *Nuclear_Fraction_Calculator*

a

Overall survival multivariate analysis		
	**P value**	**Hazard Ratio**
Stage	$<10^{-16}$	2.48 (1.8 - 3.41)
Gender	0.978	0.99 (0.63 - 1.56)
Smoking history	0.0463	0.58 (0.34 – 0.99)
SHH mRNA expression	0.0223	1.65 (1.07 – 2.52)

b

Progression free survival multivariate analysis		
	**P value**	**Hazard Ratio**
Stage	$<10^{-16}$	2.3 (1.72 – 3.09)
Gender	0.5888	1.12 (0.74 - 1.7)
Smoking history	0.8177	0.95 (0.61 - 1.48)
SHH mRNA expression	0.1719	1.29 (0.9 – 1.85)

Supplement Fig. 1 Multivariate analyses of lung adenocarcinoma patients from Kaplan-Meier Plotter database. Multivariate analysis of **a** overall survival and **b** progression free survival of lung adenocarcinoma patients is shown with stage, gender, smoking history, and *SHH* mRNA expression as variables.

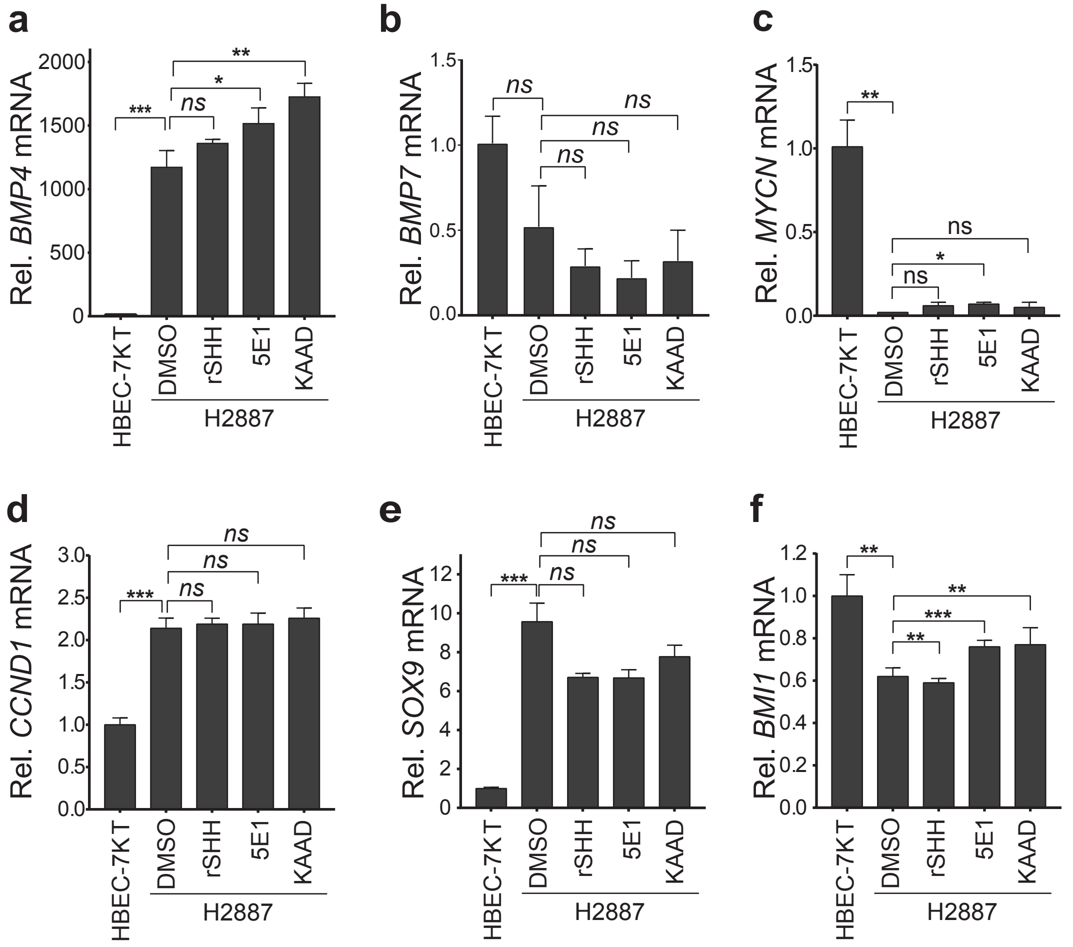

Supplement Fig. 2. Expression of Hh regulated genes in high *SHH* mRNA expressing human lung cancer cell line H2887. **a-f** H2887 cells were treated with control vehicle (DMSO), recombinant SHH (rSHH) 1μg/ml, 5E1 10 μg/ml, or KAAD-cyclopamine 300 nM for 48 hours. Expression of **a** *BMP4*, **b** *BMP7*, **c** *MYCN*, **d** *CCND1*, **e** *SOX9*, and **f** *BMI1* mRNA transcription was measured by qPCR relative to HBEC7KT cell line. The data represent mean of triplicates +/- s.d. *P< 0.05, **P< 0.01, *** P< 0.001. *ns* = not significant.

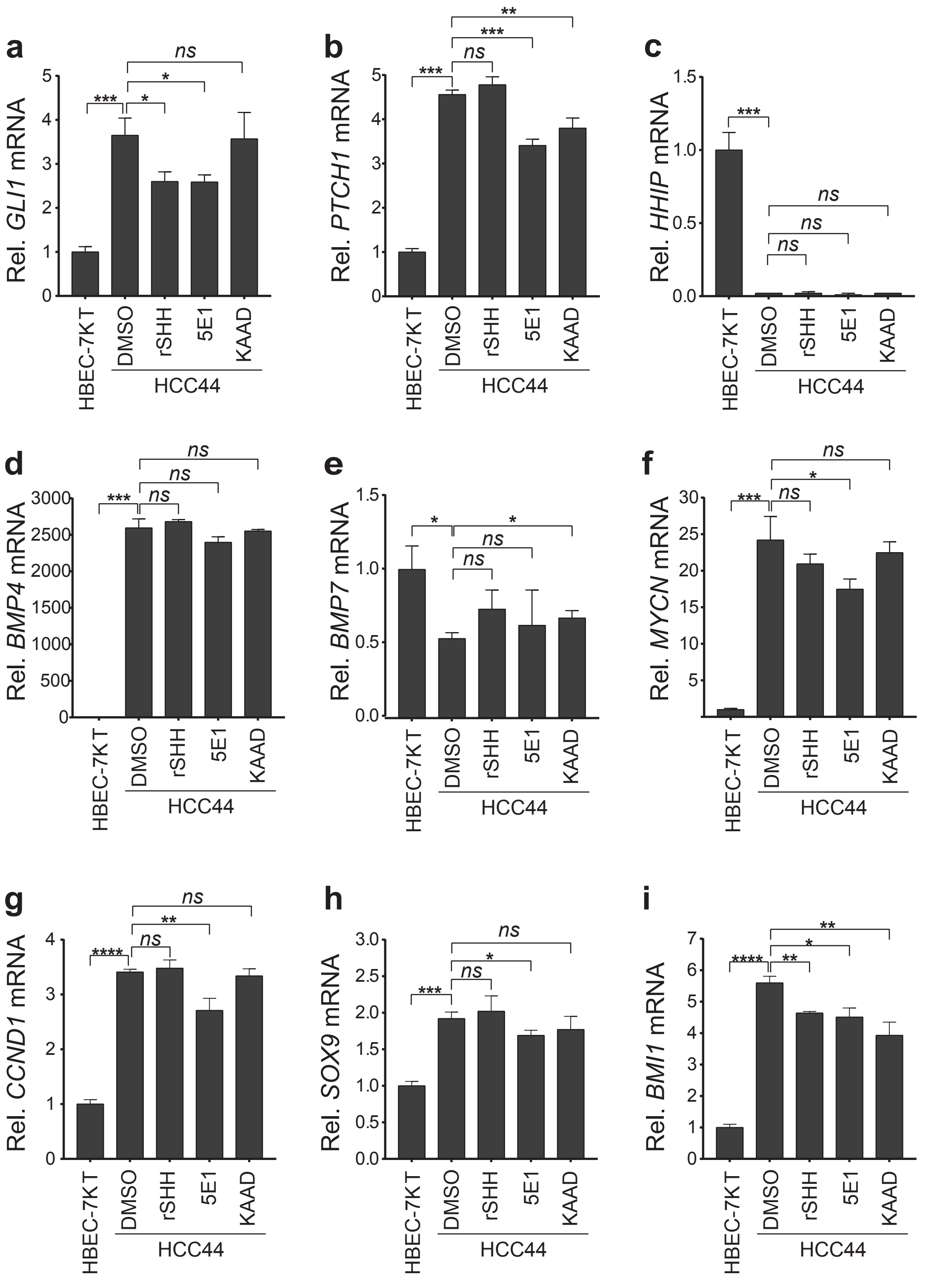

Supplement Fig. 3. Expression of Hh-pathway target genes in high *SHH* mRNA expressing human lung cancer cell line HCC44. **a-i** HCC44 cells were treated with control vehicle (DMSO), rSHH 1 μg/ml, 5E1 10 μg/ml, or KAAD-cyclopamine 300 nM for 48 hours. Expression of **a** *GLI1*, **b** *PTCH1*, **c** *HHIP*, **d** *BMP4*, **e** *BMP7*, **f** *MYCN*, **g** *CCND1*, **h** *SOX9*, and **i** *BMI1* mRNA was measured by qPCR relative to HBEC7KT cell line. The data represent mean of triplicates +/- s.d. **P*< 0.05, ***P*< 0.01, *** *P*< 0.001, **** *P*< 0.0001. *ns* = not significant.

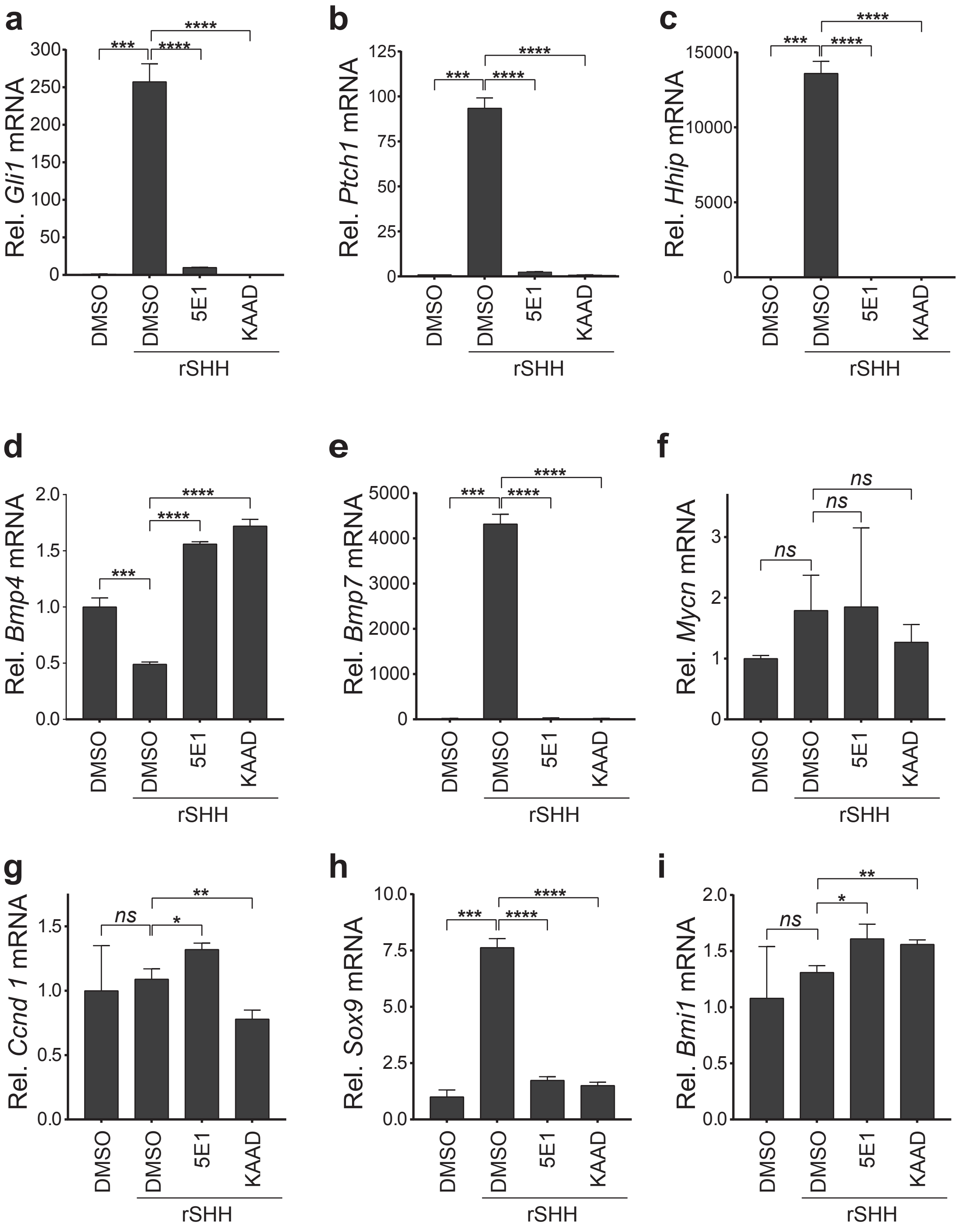

Supplement Fig. 4. Expression of Hh-pathway target genes in murine lung fibroblasts treated with recombinant SHH (rSHH). MLg lung fibroblasts were treated with DMSO control or rSHH 1 μg/ml alone or rSHH 1 μg/ml with 5E1 10 μg/ml or KAAD-cyclopamine (KAAD) 300 nM for 48 hours and then RNA was isolated and subjected to qPCR to measure the expression of **a** *Gli1*, **b** *Ptch1*, **c** *Hhip*, **d** *Bmp4*, **e** *Bmp7*, **f** *Mycn*, **g** *Ccnd1*, **h** *Sox9*, and **i** *Bmi1*. The data represent mean of triplicates +/- s.d. **P*< 0.05, ***P*< 0.01, *** *P*< 0.001, **** *P*< 0.0001. *ns* = not significant.

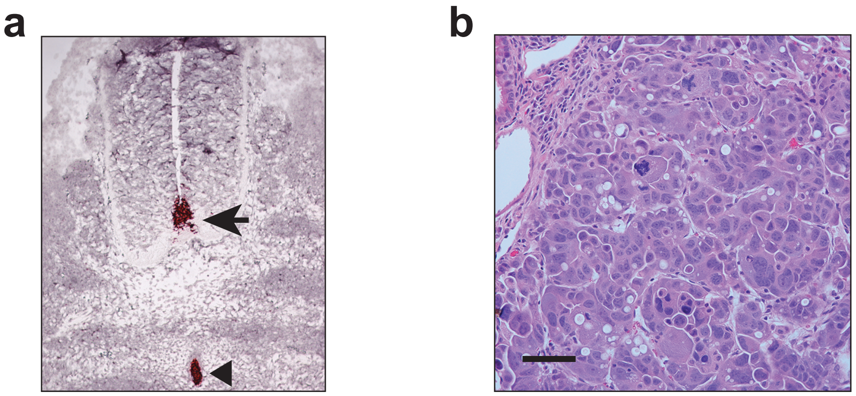

Supplement Fig. 5. *Shh* in situ hybridization. **a** Validation of *Shh* probes. E11.5 mouse embryonic neural tube is shown. RNA in situ hybridization assay was performed with RNAScope using *Shh* probes. Red puncta indicate *Shh* mRNA. Arrowhead indicates notochord and arrow indicates floor plate. **b** H&E image of tumor corresponding to RNA in situ hybridization image of Fig. 2a.   H&E image and RNA in situ hybridization image are ~55 micrometers apart. Scale bar is 50 micrometers.

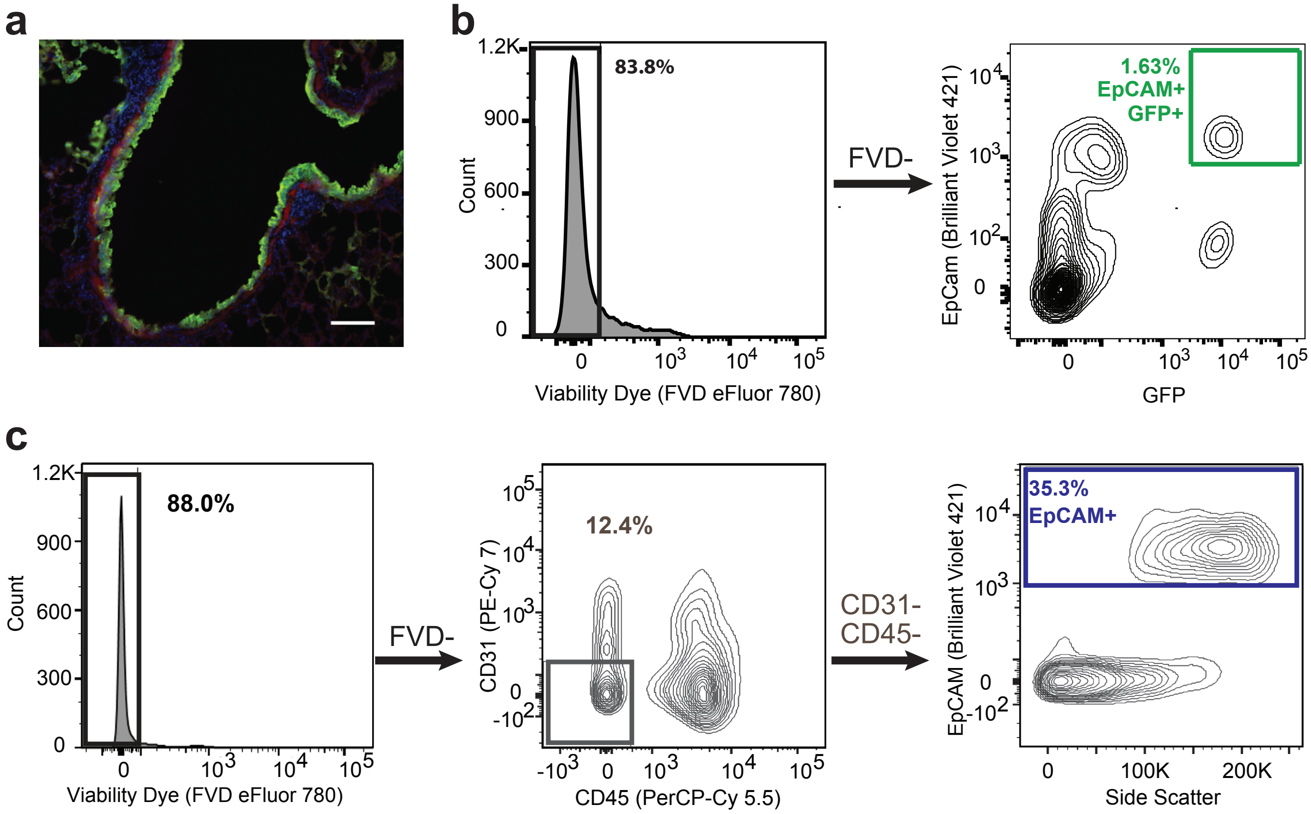

Supplement Fig. 6. FACS sorting of murine lung stromal and epithelial cells. **a** Image of lung section from *KPmTmG* mouse 4 weeks after adeno-cre infection. Adeno-cre infected cells express GFP (green) and uninfected cells express tdTomato (red). Scale bar is 100 micrometers. **b** Lungs of *KPmTmG* mice were subjected to single cell digestion 3 weeks after infection with adeno-cre. Infected lung epithelial cells (EpCAM+,GFP+) were sorted after gating on viable cells (FVD-). **c** Lungs of uninfected *KP* mice were enzymatically digested to single cells and then lung epithelial cells (EpCAM+, CD31-, CD45-) were isloated by FACS after gating on viable (FVD-) and CD31-, CD45- cells.

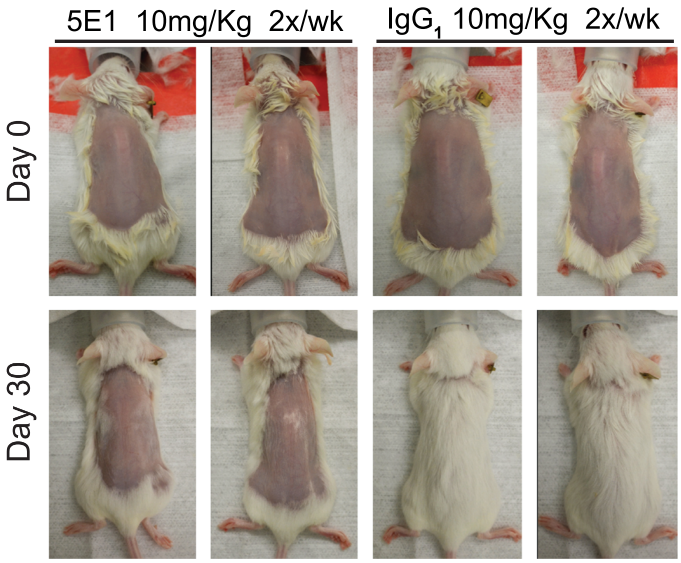

Supplement Fig. 7. Dose optimization of 5E1 antibody for *in vivo* treatment. Backs of FVB mice were shaved and hair follicles were depilated with Nair to stimulate hair regrowth. 5E1 or IgG_1 10 mg/kg i.p. twice per week were initiated immediately after hair removal for 30 days.

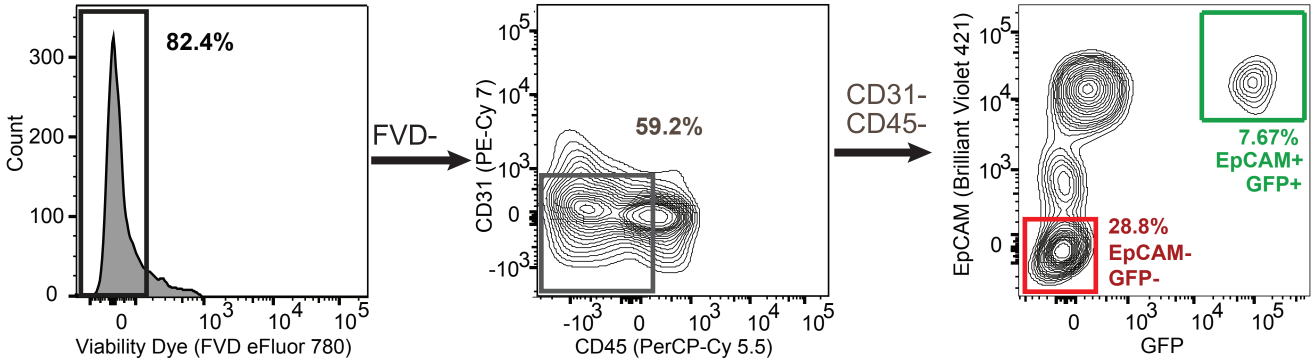

Supplement Fig. 8. FACS sorting of murine lung stromal and epithelial cells. *KPmTmG* mice were treated with 5E1 or IgG_1 for 4 weeks starting 2 weeks after adeno-cre infection. Lungs were harvested and enzymatically dissociated into single cells then stromal (CD31-, CD45-, GFP-, and EpCAM-) and epithelial (CD31-, CD45-, GFP+, and EpCAM+) cells were isloated by FACS after gating on viable cells (FVD-).

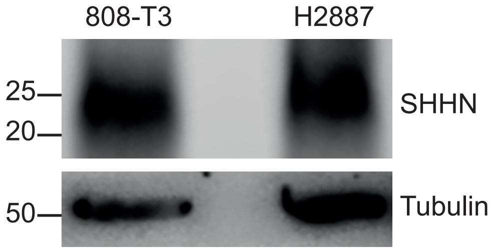

Supplement Fig. 9. SHH in *KP* adenocarcinoma cell line 808-T3. Immunoblot of active N-terminal SHH of 808-T3 cells and human high SHH expressing lung adenocarcinoma cell line H2887 is shown. Tubulin was used for loading control.

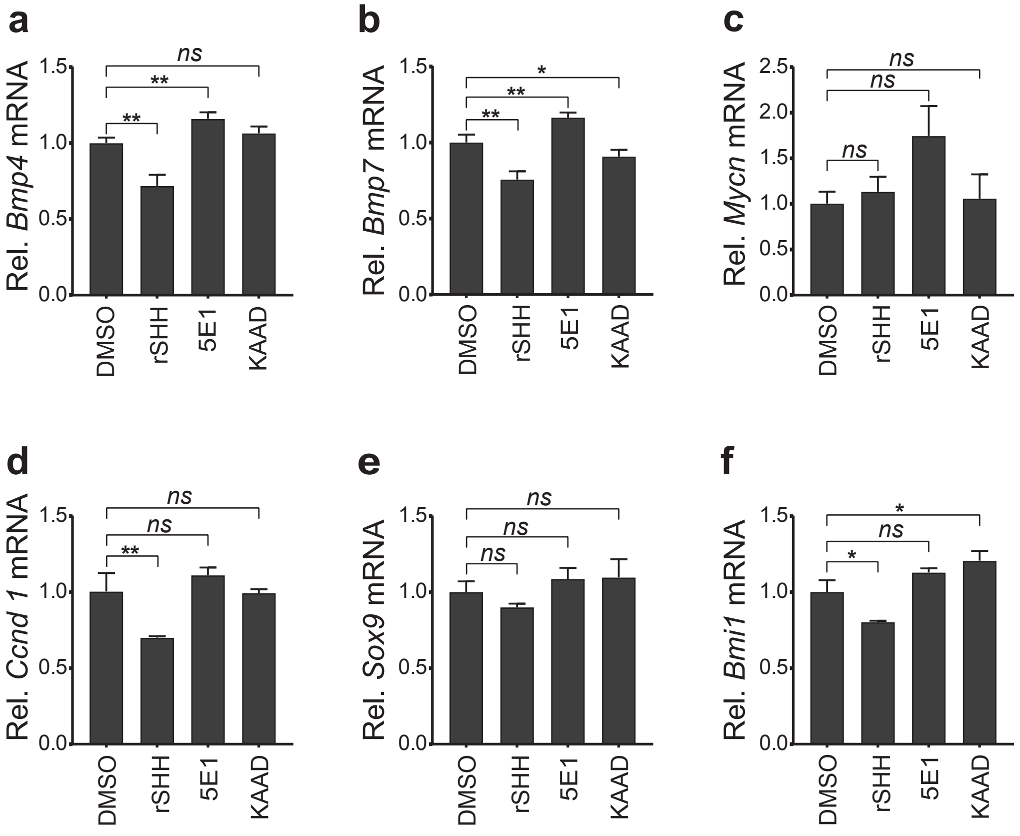

Supplement Fig. 10. Expression of Hh pathway target genes in *KP* lung adenocarcinoma cell line. 808-T3 cells were treated with DMSO vehicle rSHH 1 μg/ml, 5E1 10 μg/ml, or KAAD-cyclopamine 300 nM and then expression of **a** *Bmp4*, **b** *Bmp7*, **c** *Mycn*, **d** *Ccnd 1*, **e** *Sox9* ,and **f** *Bmi1* mRNA transcription was measured by qPCR. The data represents mean of triplicates +/- s.d. **P*< 0.05, ***P*< 0.01. *ns* = not significant.

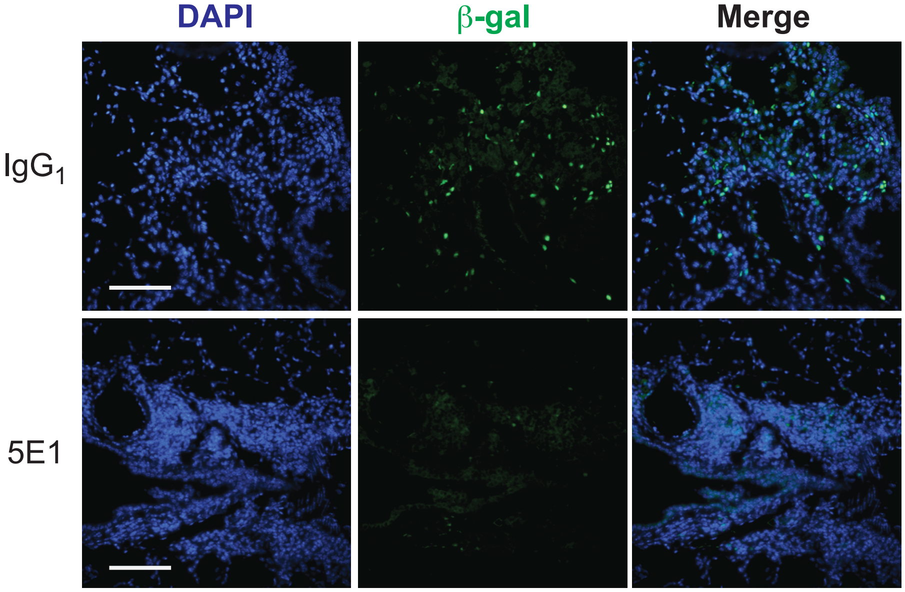

Supplement Fig. 11. Reduction of GLI1 expression with 5E1 treatment *in vivo*. *KP;Gli1*$^{LacZ/+}$ mice were treated with 5E1 or IgG_1 for 4 weeks starting 2 weeks after adeno-cre infection. Images correspond to Fig. 2h. Lung sections were stained for β-galactosidase (green) that represents GLI1. Nuclei are stained as the β-galactosidase contains a nuclear localization signal. Scale bars are 50 micrometers.

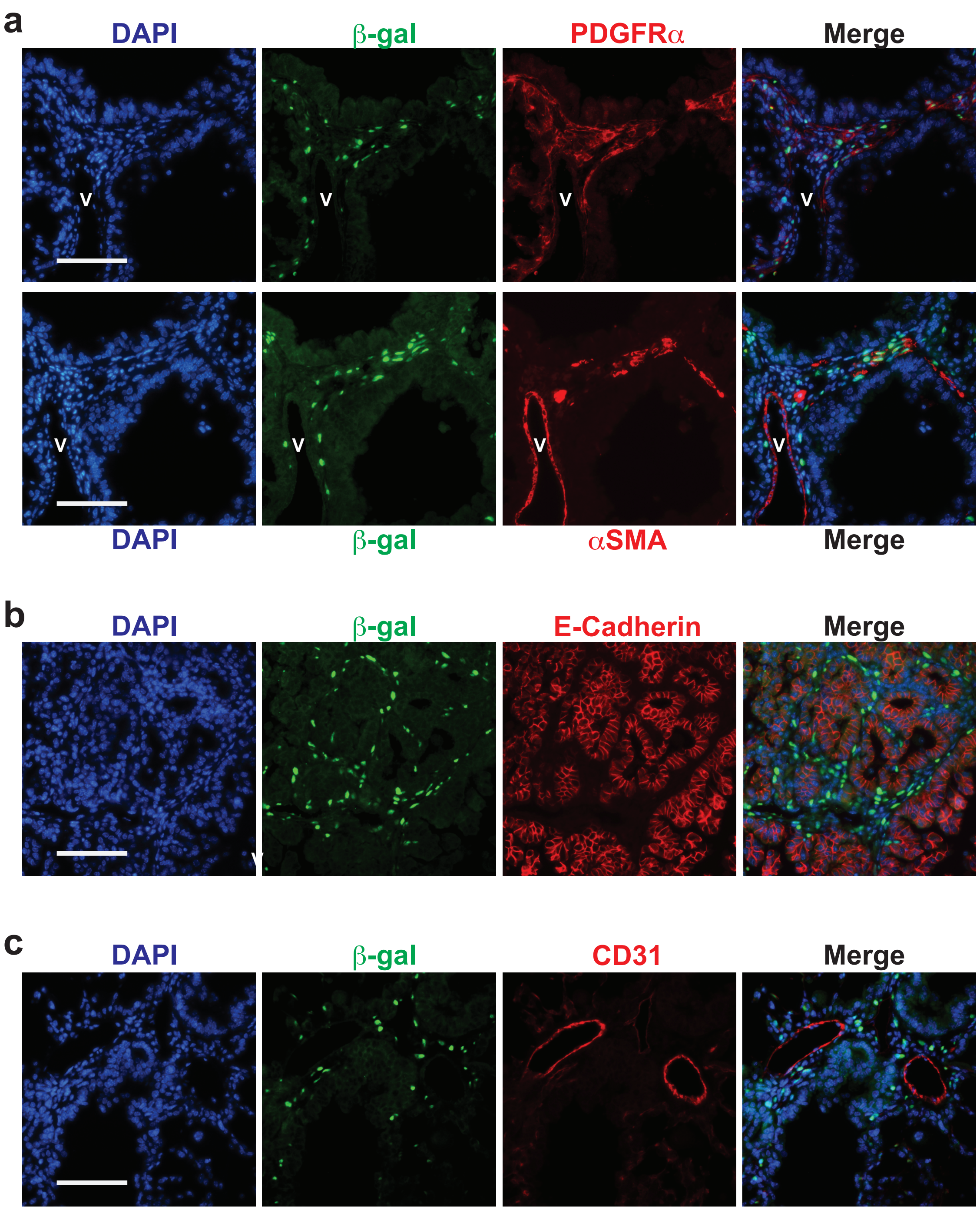

Supplement Fig. 12. Identification of GLI1 expressiong cells in murine lung. Images of lung sections from *KP;Gli1*$^{LacZ/+}$ mice 4 weeks after adeno-cre infection. Images correspond to Fig. 2i. **a** Sections ~20 micrometers apart were co-stained for β-galactosidase (green that represents GLI1) and PDGFRα (red, top panels) or αSMA (red, bottom panels). The letter 'V' indicates blood vessel. **b, c** Co-stain of β-galactosidase (green) and **b** E-Cadherin (red) or **c** CD31 (red). Scale bars are 50 micrometers.

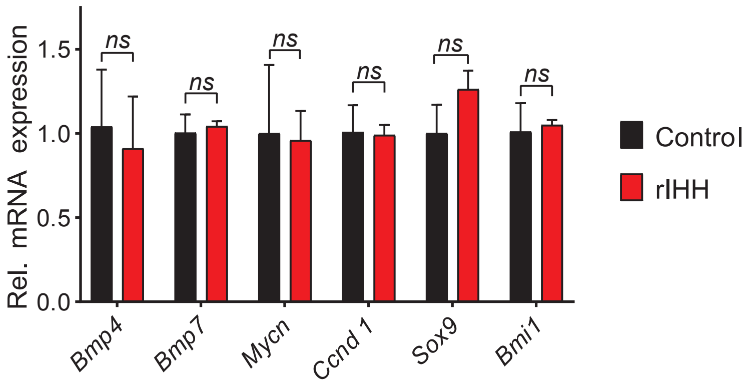

Supplement Fig. 13. Expression of Hh-pathway target genes in *KP* lung adenocarcinoma cells. 808-T3 cells were treated with rIHH 2.5 μg/ml or control vehicle for 48 hours and expression of *Bmp4*, *Bmp7*, *Mycn*, *Ccnd 1*, *Sox9* ,and *Bmi1* mRNA transcription was measured by qPCR. Data represents mean of triplicates +/- s.d. *ns* = not significant.

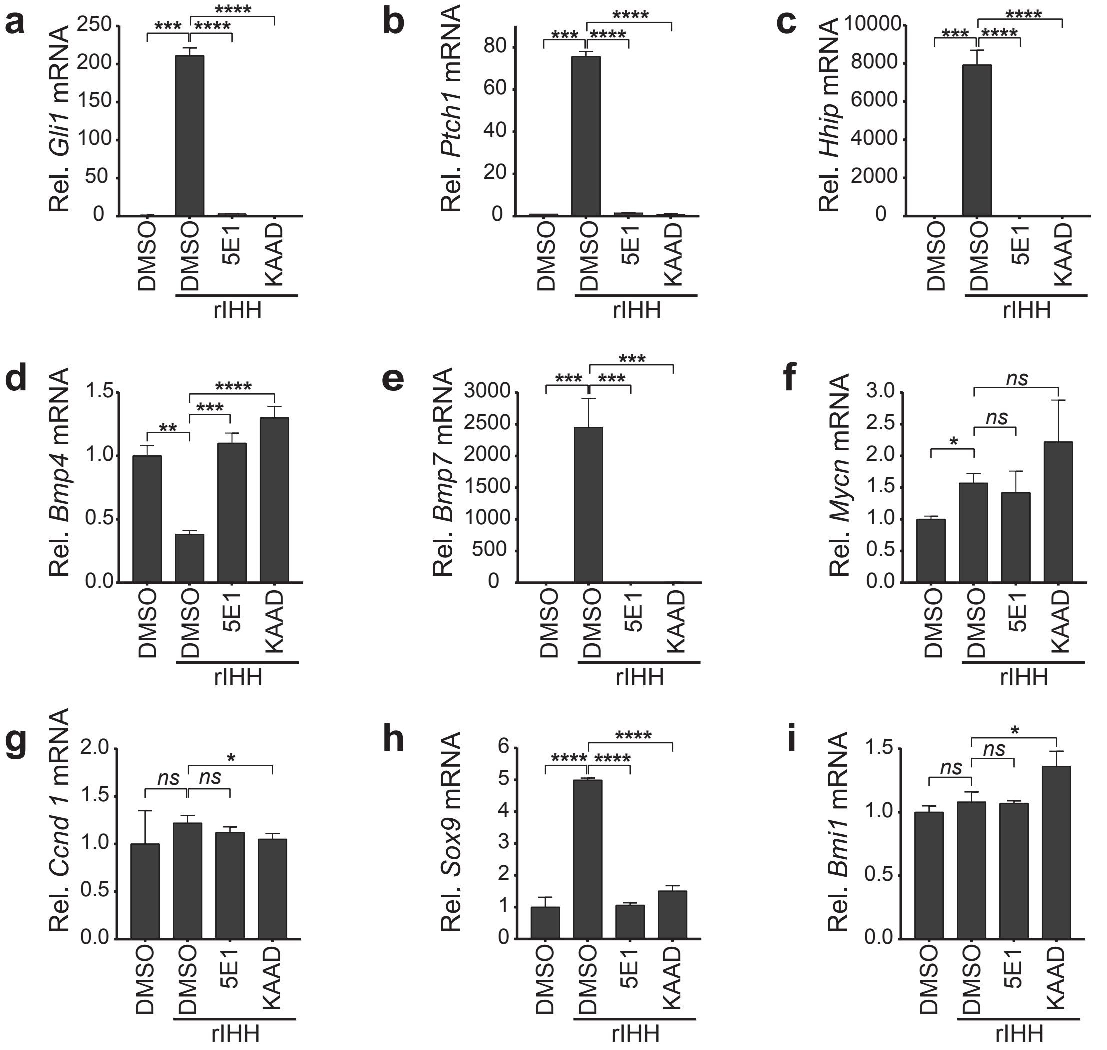

Supplement Fig. 14. Expression of Hh-pathway target genes in murine lung fibroblasts treated with recombinant IHH (rIHH). MLg lung fibroblasts were treated with DMSO control or rIHH 2.5 μg/ml alone or rIHH 1 μg/ml with 5E1 10 μg/ml or KAAD-cyclopamine (KAAD) 300 nM for 48 hr. mRNA expression of pathway target genes **a** *Gli1,* **b** *Ptch1,* **c** *Hhip,* **d** *Bmp4,* **e** *Bmp7,* **f** *Mycn,* **g** *Ccnd1,* **h** *Sox9,* and **i** *Bmi1* was measured by qPCR. The data represent mean of triplicates +/- s.d. *P< 0.05, **P< 0.01, *** P< 0.001, **** P< 0.0001. *ns* = not significant.

a

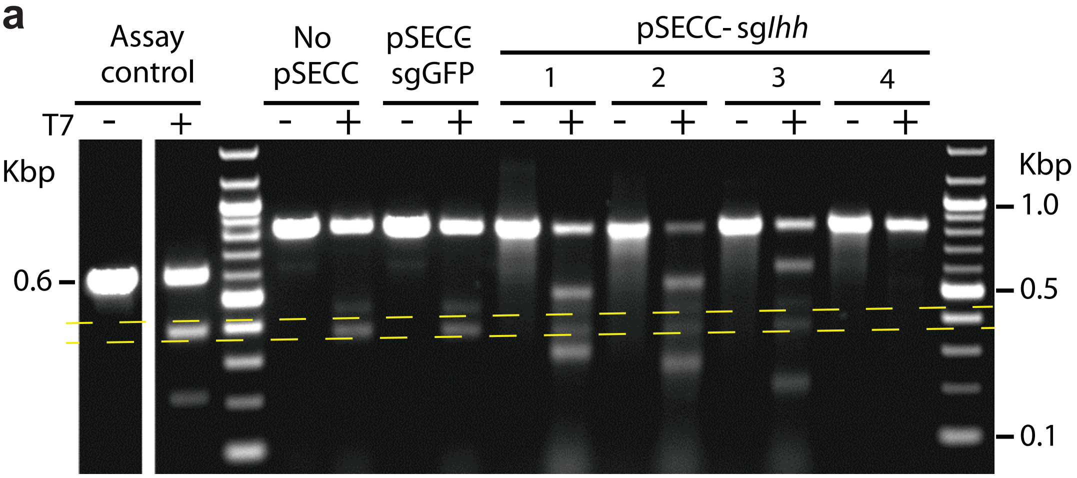

sgRNA	Expected size of digested products	Percentage of digestion
GFP	No product	0.0
1	510 & 320 bp	44.6
2	550 & 280 bp	67.4
3	610 & 220 bp	42.0
4	520 & 310 bp	0.0

b

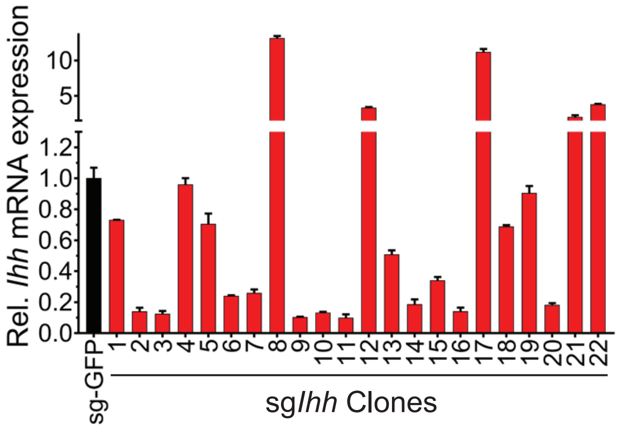

Supplement Fig. 15. Validation of candidate sgRNAs against *Ihh* (sg*Ihh*). **a** SURVEYOR assay. Green-Go cells (a reporter cell line that expresses GFP after exposure to cre-recombinase) were infected by lentiviral pSECC-Ihh or pSECC-GFP (negative control). Infected GFP+ cells were isolated by FACS, the genomic target region was amplified by RT-PCR, and subjected to SURVEYOR assay. Bands between the two yellow dashed lines represent nonspecific digested products. The table lists the expected size of the digestion products and the percent of digested bands relative to the total RT- PCR product (digested + undigested bands) for each sgRNA based on gel densitometry. **b** *Ihh* mRNA levels of clonal colonies of 808-T3 cells are shown after transient co-transfection with pSECC plasmid containing sgRNA against *Ihh* (pSECC-sg*Ihh*) or GFP (pSECC-sgGFP) and pCMV:DsRed(FRT)GFP plasmid expressing DsRed. DsRed+ transfected cells were FACS-sorted and grown as colonies after being plated at limiting dilutions. *Ihh* mRNA levels were measured by qPCR. Bulk pSECC-sgGFP transfected cells were used as control. Data represents mean of triplicates +/- s.d.

	KRAS mutation	TP53 mutation	IHH ACDbio score	% Acinar	% Lepidic	% Papillary	% Micropapillary	% Solid	Mucinous
Case 1	G13C	R248L	0	45	5	0	0	50	No
Case 2	**G12A**	**C275F**	**2+**	**10**	**70**	**0**	**5**	**15**	**Yes**
Case 3	**G12V**	**Y220C**	**1+, focal 2+**	**30**	**70**	**0**	**0**	**0**	**Yes**
Case 4	WT	WT	0	30	65	0	5	0	No
Case 5	WT	WT	0	60	30	5	5	0	No
Case 6	WT	WT	0	60	30	10	0	0	No
Case 7	**WT**	**WT**	**1+**	**30**	**70**	**0**	**0**	**0**	**Yes**
Case 8	WT	WT	0	30	70	0	0	0	No
Case 9	WT	WT	0	5	5	30	10	50	No

Supplement Fig. 16. Summary of IHH mRNA *in situ* hybridization and histological features of human lung adenocarcinoma samples. Data corresponds to Figure 5a, b. Samples that are positive for IHH mRNA are in bold.

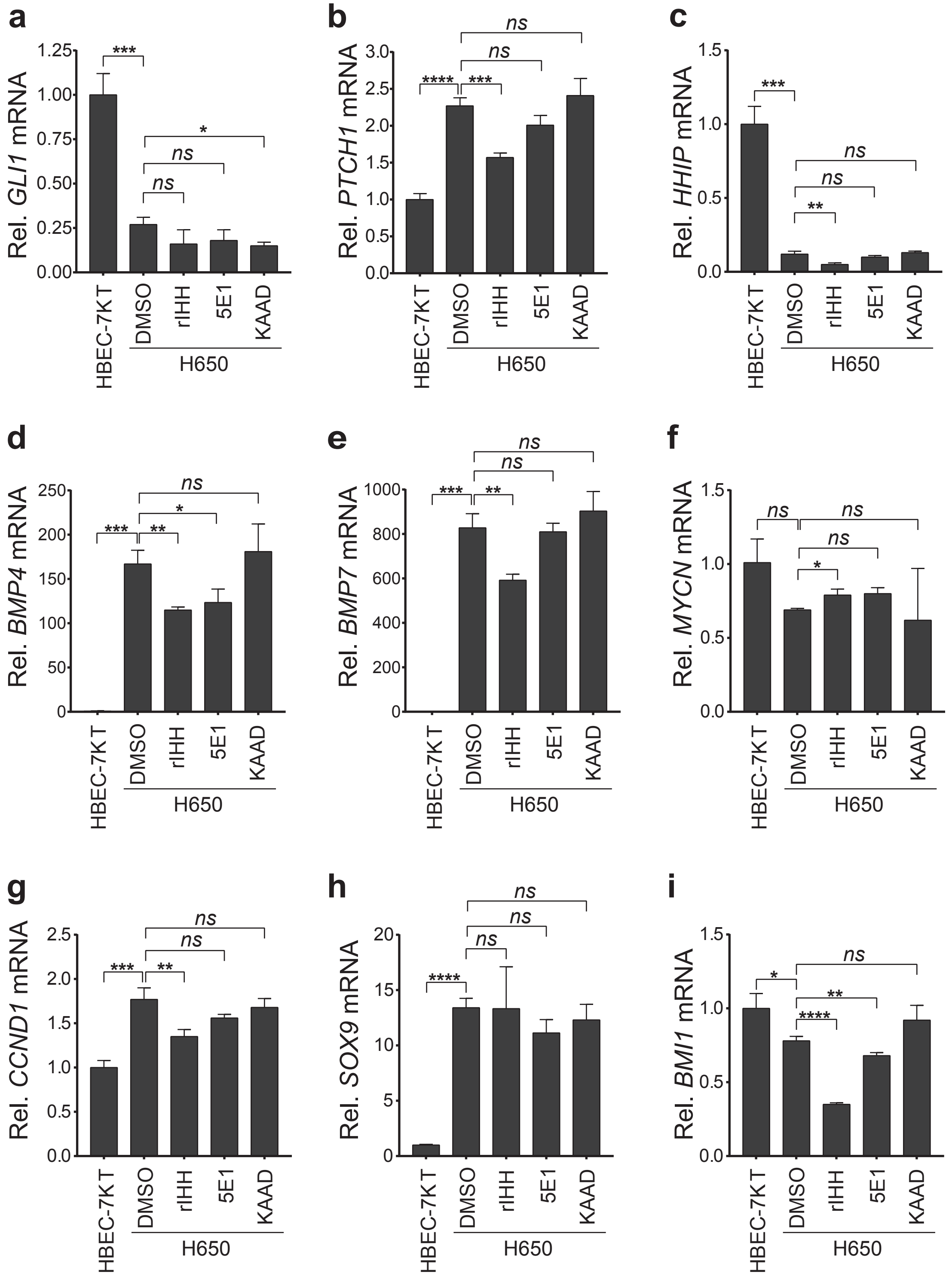

Supplement Fig. 17. Expression of Hh-pathway target genes in human lung adenocarcinoma cell line that expresses high levels of *IHH* mRNA. . **a-i** H650 LAD cells were treated with control vehicle (DMSO), recombinant IHH (rIHH) 2.5 μg/ml, 5E1 10 μg/ml, or KAAD-cyclopamine 300 nM for 48 hours. mRNA expression of pathway target genes **a** *GLI1*, **b** *PTCH1*, **c** *HHIP*, **d** *BMP4*, **e** *BMP7*, **f** *MYCN*, **g** *CCND1*, **h** *SOX9*, and **i** *BMI1* was measured by qPCR relative to HBEC7KT cell line. The data represent mean of triplicates +/- s.d. *P< 0.05, ***P*< 0.01, *** *P*< 0.001, **** *P*< 0.0001. *ns* = not significant.

Median Survival (Days)			
5E1		IgG_1	
Control	88	Control	99
NAC	97	NAC	96

Supplement Fig. 18. Median survival of mice from Figure 6f and g. Median survival of *KP* mice treated with 5E1 and NAC or vehicle control (Fig. 6f) or IgG1 with NAC or vehicle control (Fig. 6g).

Gene	Forward Primer (5’ → 3’)	Reverse Primer (5’ → 3’)
Human *HPRT1*	GGTCAGGCAGTATAATCCAAAG	GGACTCCAGATGTTTCCAAAC
Human *GAPDH*	CACCAGGGCTGCTTTTAACTC	CCTGGAAGATGGTGATGGGAT
Mouse *Gapdh*	CATTTGCAGTGGCAAAGTGGAG	ACCCCATTTGATGTTAGTGGGG
Human *SHH*	CAAGCAGTTTATCCCCAATGTG	TCACCCGCAGTTTCACTC
Mouse *Shh*	CTATGAGGGTCGAGCAGTGG	GAAACAGCCGCCGGATTTG
Human *IHH*	CCGGCTTTGACTGGGTGTAT	ATGAGCACATCGCTGAAGGT
Mouse *Ihh*	CCAGTGGCCTGGTGTGAAAC	CGTGGGCCTTGGACTCGTAA
Human *GLI1*	AGCCGTGCTAAAGCTCCAGT	CCCACTTTGAGAGGCCCATAG
Mouse *Gli1*	CAATTTCAACCCCTCCTCCTCT	AGGTGCAAAGCCAGATCCATA
Human *PTCH1*	GGACACTCTCATCTTTTGCTG	GGTAGTCTGCTTTCTGGGT
Mouse *Ptch1*	ATGCTCCTTTCCTCCTGAAACC	TGAACTGGGCAGCTATGAAGTC
Human *HHIP*	TCTCAAAGCCTGTTCCACTCA	TATGGCCTCGGCAAGTGTAAA
Mouse *Hhip*	TGAAGATGCTCTCGTTTAAGCTG	CCACCACACAGGATCTCTCC
Human *BMP4* [ref.(1)]	TGAGCCTTTCCAGCAAGTTT	CTTCCCCGTCTCAGGTATCA
Mouse *Bmp4* [ref.(2)]	TTCCTGGTAAC-CGAATGCTGA	CCTGAATCTCGGCGACTTTTT
Human *BMP7* [ref.(3)]	GCCTACTACTGTGAGGGGGAG	GAAGTAGAGGACGGAGATGGC
Mouse *Bmp7*	ACGGACAGGGCTTCTCCTAC	ATGGTGGTATCGAGGGTGGAA
Human *MYCN*	TGATCCTCAAACGATGCCTTC	GGACGCCTCGCTCTTTATCT
Mouse *Mycn*	TGTGTCTGTTCCAGCTACTGCC	ACCGCCTTGTTGTTAGAGGAGG
Human *CCND1*	CAATGACCCCGCACGATTTC	CATGGAGGGCGGATTGGAA
Mouse *Ccnd1*	AACTACCTGGACCGCTTCCTGT	GCCAGGTTCCACTTGAGCTTGT
Human *SOX9*	AGCGAACGCACATCAAGAC	CTGTAGGCGATCTGTTGGGG
Mouse *Sox9*	AGTACCCGCATCTGCACAAC	ACGAAGGGTCTCTTCTCGCT
Human *BMI1*	CGTGTATTGTTCGTTACCTGGA	TTCAGTAGTGGTCTGGTCTTGT
Mouse *Bmi1* [ref.(4)]	AGCAGCAATGACTGTGATGCACTTGAG	GCTCTCCAGCATTCGTCAGTCCATCCC

Supplement Table 1. qPCR primers used in this study.

sgRNA	Sequences (5' → 3')
GFP [ref. (5)]	GGCCACAAGTTCAGCGTGTC
Ihh-1	CTGGGTGTATTACGAGTCCA
Ihh-2	ACTGCTGGCGCGCTTAGCAG
Ihh-3	TTTACACTATGAGGGCCGCG
Ihh-4	GTAATACACCCAGTCGAAGC

Supplement Table 2. Murine sgRNA sequences used in this study.

Supplemental Material and Methods

5E1 generation and purification

5E1 Hybridoma cells (Developmental Studies Hybridoma Bank) were maintained in Hybridoma medium (Gibco) with 20% FBS (heat inactivated super low IgG serum, HyClone), 1X GlutaMax (Gibco), and 2.5 mM HEPES (Fisher Scientific). Cells were maintained for 7 days and then supernatant was collected, centrifuged, and filtered through a 0.2 μm filter. Presence of 5E1 in the supernatant was verified with an ELISA assay. 3M NaCl and 0.1M Borate were added to the supernatant and pH adjusted to 8.5 and loaded on to a protein A bead column using a peristaltic pump at 1.5 mL/min at 4°C for 3 rounds. 5E1 was eluted using ImmunoPure Genetle Ag/Ab Elution Buffer (PIERCE) and then subjected to dialysis in PBS using Slide-A-Lyzer Dialysis Cassette (Thermo Fischer Scientific).

RNA in situ hybridization analysis of human lung adenocarcinoma samples

RNA *In situ* hybridization (ISH) was conducted by automated RNAscope assay using the Leica Bond RX autostainer (Leica Biosystems, Nussloch, GmbH) to visualize single RNA molecule per cell as a single dot in FFPE tissue samples at least. The procedure performed on BOND RX system are briefly described as the following. Tissue sections of 5um thick were deparaffinized and rehydrated, then followed with the Leica Bond prestaining and staining protocols. Antigen retrieval was performed with ER2 (BOND Epitope Retrieval Solution 2) at 95°C for 15 minutes and ACD Protease treatment at 40°C for 15 min; then RNA-specific probes were hybridized to target RNA for 120 min. The signals were amplified by multiple steps, which was followed by hybridization to horseradish peroxidase (HRP)-labeled probes and detection using the 3,3′-Diaminobenzidine (DAB) chromogenic substrate. After that, cell nuclei were counterstained with hematoxylin. The RNAscope probes assayed in this study were: IHH (target probe) (RNAscope® LS 2.5 Probe- Hs-IHH, 472388, Advanced Cell Diagnostic, Hayward, CA, USA), PPIB

(housekeeping gene, positive control probe) (Hs-PPIB, 313908, Advanced Cell Diagnostic, Hayward, CA, USA) and dapB (bacterial gene, negative control probe) (312038, Advanced Cell Diagnostic, Hayward, CA, USA). Positive and negative control probes were used to assess RNA quality of tissue section and optimal permeabilization.

RNA expression of IHH was scored using conventional bright-field microscopy in adenocarcinoma malignant cells. We applied a semi-quantitatively scoring system, in a scale of 0-4, based on the number of RNA copies per cell (0, no staining or <1 dot/10 cells; 1+, 1-3 dots/cell; 2+, 4-9 dots per cell, None or very few dot clusters; 3+, 10-15 dots/ cell and <10% dots are in clusters; 4+, >15 dots/cell and >10% dots are in clusters,). Positive (PPIB) and negative (Dapb) control probes were also evaluated, dapB score of <1 and PPIB score ≥2 with relatively uniform PPIB signals throughout the sample were considered adequate for analysis (data not shown).

Lentivirus generation

Lentivirus containing pSECC-Ihh and pSECC-GFP were generated as described previously with some modifications (6). Briefly, 85-90% confluent 293-T cells in 15 cm cell culture dish were co-transfected with 16 μg pSECC-*Ihh* or pSECC-GFP, 8 μg pCMV-dR8.91 (packaging vector), and 4 μg pCMV-VSV-G (enveloping vector) using Lipofectamine 3000 (Thermo Fischer Scientific). pCMV-dR8.91 and pCMV-VSV-G vectors were kindly provided by Dr. John Minna (UTSW). Growth medium was replaced after 16 hours, subsequent supernatant was collected 48 hours later and centrifuged at 800xg at 4°C to precipitate cell debris. Supernatant above the cell debris pellet was collected and ultracentrifuged at 100,000xg for 2 hours. Subsequent pellet containing the lentivirus was resuspended in OptiMEM (Gibco). Titration of lentivirus was performed per previously described protocol (https://tinyurl.com/yd2qk9l6).

SURVEYOR assay

Lentivirus containing selected pSECC-*Ihh* was generated on a small scale (10 cm cell culture plate) using 293T cells as described above. Cell culture supernatant (containing lentivirus) was collected 72 hours after transfection and placed on 10 cm cell culture dish of 70-80% confluent Green-Go reporter cells and maintained for 48 hours. GFP+ Green-Go cells (6) were isolated by FACS. Genomic DNA from GFP+ cells was isolated using PureLink Genomic DNA Mini Kit (ThermoFischer Scientific). The sg-*Ihh* target region of genomic DNA was amplified by PCR using the following primers: forward 5'-GTCCCATGAGTGCTGTCGA-3' and reverse 5'-TGACCTGCATTTGCGTGGTA-3'. Mutation detection on PCR product was performed using EnGen Mutation Detection Kit (New England BioLabs) and following manufacturer instructions.

Analysis of γH2AX+ nuclei in immunohistochemical stained lung sections

Slides with immunohistochemically stained lung sections were scanned at 40x magnification using a Hamamatsu Nanozoomer. The scanned images were exported as tiff images at 10x resolution. Analysis was performed on Fiji imaging software. Tumors were outlined interactively using the custom "*ROI_Draw*" macro. To obtain the fraction of γH2AX positive nuclei in tumors, a custom macro, "*Nuclear Fraction Calculator"*, was used. Briefly, "*Nuclear_Fraction_Calculator"* macro operates as follows. DAB positive nuclei were segmented using the "Colour Deconvolution" plugin written by Gabriel Landini (Image>Color>Colour Deconvolution in Fiji or as an ImageJ plugin available from (https://tinyurl.com/yckvnp3f). The built-in H DAB matrix is used to separate DAB staining from hematoxylin staining, the DAB channel is converted to 8-bit grayscale, the contrast is inverted, and a binary mask is generated by thresholding the image using the maximum entropy method, giving the user an option to adjust the threshold interactively. A watershed operation is used to separate connected nuclei and then ultimate

points is used to represent each object with a dot. To segment the total nuclei, the original color image is converted to 8-bit grayscale and the "Mexican Hat Filter" plugin by Dimiter Prodanov (https://tinyurl.com/y7vmzrow) was used to smooth the image and enhance the edges of the nuclei. A binary mask is made, followed by watershed and ultimate points to mark each object. The previously stored ROI set is used together with these masks for analysis of each tumor to obtain the area of the tumor, the number of DAB stained (γH2AX+) nuclei, the total number of nuclei and the fraction of DAB stained (γH2AX+) nuclei. "*ROI_Draw*" and "*Nuclear_Fraction_Calculator"* macros are compatible with ImageJ and Fiji.

Supplemental References

1. Kestens C, Siersema PD, Offerhaus GJ, van Baal JW. BMP4 Signaling Is Able to Induce an Epithelial-Mesenchymal Transition-Like Phenotype in Barrett's Esophagus and Esophageal Adenocarcinoma through Induction of SNAIL2. PLoS One. 2016;11(5):e0155754. Epub 2016/05/19.

2. Liu Y, Du SY, Ding M, Dou X, Zhang FF, Wu ZY, et al. The BMP4-Smad signaling pathway regulates hyperandrogenism development in a female mouse model. J Biol Chem. 2017;292(28):11740-50. Epub 2017/06/03.

3. Kodama A, Yoshino O, Osuga Y, Harada M, Hasegawa A, Hamasaki K, et al. Progesterone decreases bone morphogenetic protein (BMP) 7 expression and BMP7 inhibits decidualization and proliferation in endometrial stromal cells. Hum Reprod. 2010;25(3):751-6. Epub 2010/01/21.

4. Leung C, Lingbeek M, Shakhova O, Liu J, Tanger E, Saremaslani P, et al. Bmi1 is essential for cerebellar development and is overexpressed in human medulloblastomas. Nature. 2004;428(6980):337-41. Epub 2004/03/19.

5. Shalem O, Sanjana NE, Hartenian E, Shi X, Scott DA, Mikkelson T, et al. Genome-scale CRISPR-Cas9 knockout screening in human cells. Science. 2014;343(6166):84-7.

6. Sanchez-Rivera FJ, Papagiannakopoulos T, Romero R, Tammela T, Bauer MR, Bhutkar A, et al. Rapid modelling of cooperating genetic events in cancer through somatic genome editing. Nature. 2014;516(7531):428-31.
